# The effect of sprint interval training on key cardiometabolic risk factors in children and adolescents: a systematic review and meta-analysis

**DOI:** 10.3389/fphys.2025.1694357

**Published:** 2025-12-18

**Authors:** Weibao Liang, Jianmin Dai, Chuannan Liu, Xujie Yan, Shuting Xu, Wenbai Huang

**Affiliations:** 1 School of Physical Education, Jinan University, Guangzhou, China; 2 Guangdong Provincial Key Laboratory of Speed Capability Research, Su Bingtian Center for Speed Research and Training, Jinan University, Guangzhou, China; 3 College of Sports Science, Kyungnam University, Changwon, Republic of Korea; 4 School of Physical Education and Health, Zunyi Medical University, Zunyi, China; 5 School of Physical Education and Sports Science, Hengyang Normal University, Hengyang, China; 6 The First Affiliated Hospital of Jinan University, GuangZhou, China

**Keywords:** sprint interval training, moderate-intensity continuous training, high-intensity interval training, adolescents, cardiometabolic health, cardiorespiratory fitness

## Abstract

**Background:**

Although sprint interval training (SIT) is a time-efficient modality known to improve adult cardiometabolic health, a comprehensive synthesis of its effects in pediatric populations is lacking.

**Objective:**

This systematic review and meta-analysis evaluated the impact of SIT on key cardiometabolic risk factors in children and adolescents.

**Methods:**

Six electronic databases were searched for trials comparing SIT against non-exercising control (CON) or moderate-intensity continuous training (MICT). A random-effects model was used to compute the standardized mean difference (SMD). The study quality and evidence certainty were assessed using the Cochrane RoB 2 and GRADE frameworks, respectively.

**Results:**

Fourteen studies with 467 participants were included. Compared to CON, SIT elicited a large, significant improvement in cardiorespiratory fitness (VO_2_max) (number of studies, k = 12; SMD = 1.43, *p* = 0.004) based on evidence of moderate certainty. Significant improvements in body composition were also observed, including a large reduction in the body fat percentage (k = 7; SMD = −0.83, *p* = 0.012), a moderate reduction in waist circumference (k = 9; SMD = −0.69, *p* = 0.006), and a small reduction in body weight (k = 12; SMD = −0.15, *p* = 0.020). In contrast, SIT had no significant effects on the blood lipids, glycemic control parameters, or blood pressure (low to very low certainty evidence). Direct comparisons between SIT and MICT revealed no significant differences for any outcome.

**Conclusion:**

SIT is an effective strategy for enhancing cardiorespiratory fitness and improving body composition in children and adolescents. With an efficacy comparable to that of traditional MICT, it represents a viable, time-efficient exercise alternative for pediatric populations.

**Systematic Review registration:**

https://www.crd.york.ac.uk/PROSPERO/view/CRD420251131717, identifier CRD420251131717.

## 1 Introduction

The developmental stages of childhood and adolescence represent a critical period for establishing lifelong health trajectories and preventing chronic diseases. Nevertheless, the escalating global prevalence of sedentary behaviors has led to insufficient levels of physical activity (Global status, 2022) and a sustained increase in the rates of overweight and obesity among this demographic (World Obesity Federation, 2023). These adverse epidemiological trends significantly elevate the prospective risk for developing cardiometabolic conditions, including type 2 diabetes mellitus, hypertension, and cardiovascular disease, later in life (Jaacks et al., 2019). Consequently, promoting effective, feasible, and engaging exercise interventions for pediatric populations has become a paramount priority in global public health.

Historically, moderate-intensity continuous training (MICT), which is exemplified by 30 min–60 min of sustained aerobic exercise, has been regarded as the standard intervention for enhancing cardiorespiratory fitness and promoting health (O’Donovan et al., 2010). Although the benefits of MICT are well-documented, the substantial time commitment required by such protocols often constitutes the principal barrier to habitual participation in physical activity among children and adolescents (Biddle and Asare, 2011).

In recent years, considerable scholarly attention has been directed toward sprint interval training (SIT) as a time-efficient alternative. SIT is characterized by brief (≤30 s), repeated bouts of maximal-effort exercise interspersed with extended periods of recovery. A substantial body of evidence from adult populations indicates that SIT protocols, often requiring a total time commitment of only 10 min–20 min per session, can elicit improvements in cardiorespiratory fitness and glycemic regulation that are comparable to and, in some instances superior, to those achieved with MICT protocols of considerably greater duration (Gibala et al., 2012; Weston et al., 2014). This characteristic presents a promising strategy for addressing the pervasive barrier of insufficient time.

However, caution is required when extrapolating the findings from adult populations to youth, who undergo unique physiological and psychological maturation. Pediatric populations differ from their adult counterparts with respect to energy metabolism, cardiovascular adaptations, and their perceptual and recuperative responses to high-intensity exercise (Armstrong and McManus, 2011). Although several preliminary investigations have explored the application of SIT in pediatric cohorts, their findings have been inconsistent, with studies often limited by small sample sizes and heterogeneous intervention designs. For instance, while some studies have reported significant enhancements in cardiorespiratory function, conclusions regarding improvements in metabolic markers, such as blood lipids or insulin sensitivity, remain equivocal.

In the last 5 years, research progress in this field has largely focused on high-intensity interval training (HIIT) broadly. Several key meta-analyses have synthesized the benefits of general HIIT on various health outcomes in pediatric populations, including cardiometabolic risk factors (Liu et al., 2020; Cao et al., 2021; 2022; Wang et al., 2024; Zheng et al., 2025), cardiorespiratory fitness (Deng and Wang, 2024), and in specific contexts such as schools (Duncombe et al., 2022).

However, there is a critical shortcoming and controversy. These valuable reviews utilize a “lumping” approach; they analyze all forms of high-intensity training together. Their PICO criteria often combine protocols such as long-interval submaximal HIIT (e.g., 4 min at 85%–95% HR_max_) with the very specific intervention we are focused on: SIT. SIT is a unique physiological stimulus characterized by extremely brief (≤30 s) “all-out” or “supramaximal” efforts.

The shortcoming of this “lumping” is that it may obscure the true, isolated effects of SIT as a distinct, highly time-efficient modality (Cao et al., 2021). The key controversy/gap in the field is, therefore, whether the specific effects of SIT are “diluted” by the average effects of general HIIT. The significance of this study is that it is the first meta-analysis globally to specifically isolate and independently quantify the effects of SIT on cardiometabolic risk factors in youth, thereby addressing this critical gap.

Accordingly, the primary objective of this investigation was to conduct the first comprehensive and quantitative evaluation of the effects of SIT, as a distinct modality, on key cardiometabolic risk factors in children and adolescents through a systematic review and meta-analysis. The specific aims were as follows: 1. to determine the absolute efficacy of SIT by comparing its effects against non-exercising control conditions and 2. to assess its relative efficacy by comparing its effects against those of traditional MICT.

## 2 Methods

The conduct and reporting of this systematic review and meta-analysis adhered strictly to the Preferred Reporting Items for Systematic Reviews and Meta-Analyses (PRISMA) 2020 statement (Page et al., 2021). The protocol for this study was pre-registered on the International Prospective Register of Systematic Reviews (PROSPERO) under the registration number: CRD420251131717.

## 3 Data sources and searches

We performed a systematic search across six electronic databases, namely, PubMed, Embase, Web of Science, Scopus, the Cochrane Library, and SPORTDiscus, from their inception to June 2025. The search strategy incorporated a combination of keywords and subject headings relevant to the population, intervention, and study design.

### 3.1 Study eligibility criteria

We predicated the inclusion of studies upon the population, intervention, comparator, and outcomes (PICO) framework.

Population: The study population comprised children and adolescents aged 6–18 years, including healthy-weight, overweight/obese, and clinical cohorts.

Intervention: The intervention of interest was SIT, which is broadly defined as any form of intermittent exercise explicitly designated as SIT by the original authors or described as involving “all-out” or “supramaximal” intensity efforts. Protocols described as HIIT that used submaximal intensities (e.g., 85%–95% HRmax) were explicitly excluded to isolate the effects of ‘all-out’ sprinting.

Comparator: Eligible comparators included at least one of the following: 1. a non-training or standard physical education control group (CON); 2. a moderate-intensity continuous training group (MICT).

Outcomes: Studies were required to report at least one quantitative measure of cardiometabolic health or physical fitness, with a primary focus on cardiorespiratory fitness (e.g., VO_2_max), body composition, blood lipids, glycemic control, and blood pressure.

Study design: All interventional study designs, including randomized controlled trials (RCTs) and non-randomized controlled trials (non-RCTs), were deemed eligible for inclusion.

Based on these criteria, two independent reviewers (CL and XY) screened all the study titles and abstracts. Studies assessed as relevant or unclear were subjected to full-text review. Discrepancies were resolved by a third independent reviewer (WH).

### 3.2 Data extraction

A standardized data extraction form was utilized to collect information from each included study. Two reviewers (WL and SX) independently extracted the data. Data extraction was performed in duplicate utilizing a standardized pro forma designed to capture the study characteristics, participant demographics, intervention protocols, and outcome data (i.e., the sample size, mean, and standard deviation at baseline and post-intervention).

### 3.3 Risk of bias assessment

We utilized the Cochrane risk of bias 2 (RoB 2) tool for the quality assessment of the included studies (Higgins et al., 2011). This assessment was performed independently by two reviewers (CL and XY), with any discrepancies being resolved by a third independent reviewer (WH).

Each study was evaluated across the five standard RoB 2 domains: 1. bias arising from the randomization process; 2. bias due to deviations from intended interventions; 3. bias due to missing outcome data; 4. bias in the measurement of the outcome; and 5. bias in the selection of the reported result.

Based on this evaluation, each study was assigned an overall risk-of-bias rating of ‘low risk,’ ‘some concerns,’ or ‘high risk.’

### 3.4 Statistical analyses

The primary effect measure for this analysis was the standardized mean difference (SMD), which was calculated as Hedges’ g within a random-effects model framework. We computed the mean changes and the standard deviations of those changes from baseline under the assumption of a pre–post correlation coefficient of 0.8. We assessed heterogeneity using Cochran’s Q test and the I^2^ statistic and planned for the execution of subgroup and leave-one-out sensitivity analyses. The certainty of the body of evidence was formally rated using the GRADE approach. All statistical analyses were performed using R (version 4.5.1). Orchard plots were used for visualizing meta-analytic effects and were generated using the orchaRd package (version 2.0) (Nakagawa et al., 2023).

## 4 Results

### 4.1 Study selection

**FIGURE 1:**
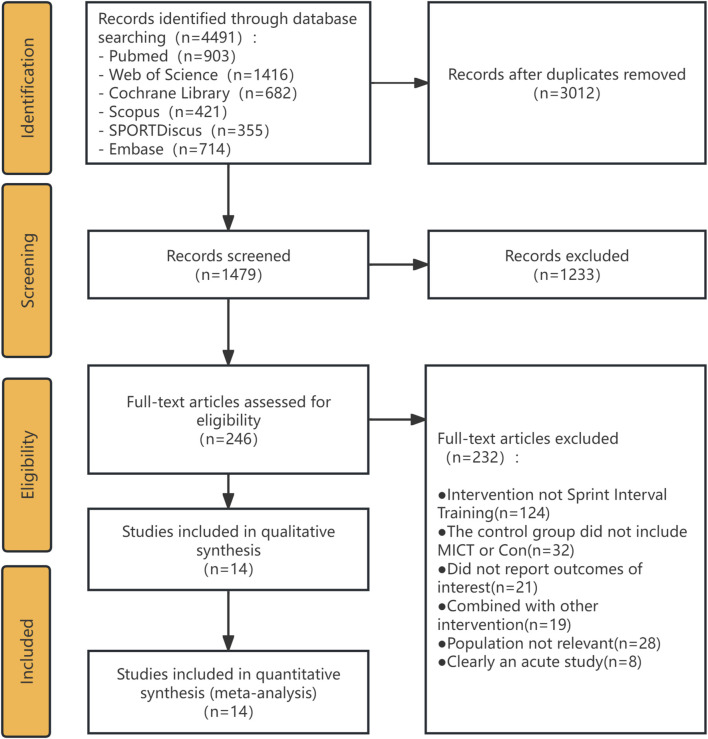
PRISMA flow diagram detailing the study selection process.

The literature search and study selection process is delineated in the PRISMA flow diagram (Figure 1). Our initial systematic search across six databases yielded 4,491 records. Following the removal of 3,012 duplicates, the titles and abstracts of the remaining 1,479 records were screened. At this stage, 1,233 records were excluded, primarily because they were reviews, commentaries, or conference abstracts or were deemed irrelevant based on the population, intervention, or outcomes described in the title and abstract. This left 246 articles for full-text evaluation. After a detailed review of the full texts, a further 232 articles were excluded. As detailed in Figure 1, the primary reasons for exclusion at this stage were incorrect intervention (not SIT) (n = 124), ineligible control group (did not include MICT or Con) (n = 32), non-relevant population (n = 28), did not report outcomes of interest (n = 21), combined with other intervention (n = 19), and clearly an acute study (n = 8). Ultimately, 14 studies (Rosenkranz et al., 2012; Racil et al., 2013; Boer et al., 2014; Lau et al., 2015; Martin et al., 2015; Lazzer et al., 2017; Chuensiri et al., 2018; Martin-Smith et al., 2019; Taylor et al., 2019; Meng et al., 2022; Salus et al., 2022b; 2022a; Tadiotto et al., 2023; Su et al., 2025) that met all the predefined inclusion criteria were included in this systematic review and meta-analysis.

### 4.2 Characteristics of the studies

#### 4.2.1 Participant characteristics

A summary of the participant characteristics from the 14 included studies is presented in Table 1. These studies, published between 2012 and 2025, collectively included 467 children and adolescents. The study populations were diverse: nine studies focused on children and adolescents who were overweight or obese, two investigated adolescents with special health conditions (including intellectual disabilities and severe mental illness), and the remaining three recruited healthy pediatric populations. The mean age of the participants across all studies ranged from 9.1 ± 1.2 to 17.3 ± 3.1 years. Regarding sex distribution, five studies exclusively enrolled male candidates, two enrolled only female candidates, and seven included mixed-sex cohorts. The majority of the participants did not engage in regular physical activity prior to the intervention. As shown in Table 1, the terminology used to describe participant activity levels varied across studies; specific definitions are provided in the Table 1 notes.

**TABLE 1:** Characteristics of the included studies.

Study	Population	Physical activity level	N	Men ratio (%)	Age (years)	BMI (kg/m^2^)
Boer et al. (2014)	Adolescents with intellectual disability	NR	46	65.2	17.3 ± 3.1	27.5 ± 3.7
Chuensiri et al. (2018)	Obese preadolescent boys	No regular exercise	26	100	10.8 ± 0.3	25.7 ± 1.2
Lau et al. (2015)	Overweight children	NR	48	75	10.4 ± 0.9	23.6 ± 2.6
Lazzer et al. (2017)	Severely obese adolescent boys	Inactive	30	100	16.4 ± 1.0	37.1 ± 3.5
Martin et al. (2015)	Healthy Scottish adolescents	Standard PE students	37	83.8	16.8 ± 0.5	22.2 ± 2.8
Martin-Smith et al. (2019)	Healthy adolescents	Recreationally active	52	61.5	16.9 ± 0.4	22.1 ± 2.3
Racil et al. (2013)	Obese adolescent female individuals	No systematic exercise	34	0	15.9 ± 0.3	30.8 ± 1.6
Rosenkranz et al. (2012)	Non-asthmatic prepubescent children	Inactive	16	12.5	9.1 ± 1.2	18.9 ± 4.0
Salus et al. (2022a)	Adolescent boys with obesity	No regular exercise	28	100	13.4 ± 0.4	31.5 ± 1.3
Salus et al. (2022b)	Adolescent boys with obesity	No regular exercise	28	100	13.4 ± 0.4	31.5 ± 1.3
Su et al. (2025)	Healthy male adolescents	Untrained	24	100	13.1 ± 0.9	21.7 ± 4.5
Tadiotto et al. (2023)	Overweight adolescents	No regular exercise	52	50	14.0 ± 1.4	2.1 ± 0.8 (z-score)
Taylor et al. (2019)	Adolescents with serious mental illness	Inactive	30	36.7	16.0 ± 1.2	25.7 ± 6.2
Williams et al. (2022)	Adolescent girls	NR	16	0	11.7 ± 0.3	18.2 ± 2.6

Data are presented as the mean ± standard deviation (SD) or count (%). BMI, body mass index; N, total number of participants included in the final analysis; NR, not reported; PE, physical education; SMI, serious mental illness. Terms for physical activity level (e.g., inactive and untrained) were extracted directly from the included studies. Based on the context of the original studies, ‘inactive’ or ‘sedentary’ generally refers to participants not meeting recommended physical activity guidelines. ‘Untrained’ typically refers to participants not engaged in a systematic or sport-specific training regimen, though they were not necessarily completely inactive.

#### 4.2.2 Intervention characteristics

The intervention protocols of the 14 included studies are detailed in Table 2. The total duration of the interventions varied considerably, ranging from a minimum of 2 weeks to a maximum of 15 weeks. The training frequency for the SIT groups was typically 2–3 sessions per week. It should be noted that the ‘session duration’ listed in Table 1 (e.g., 30 min–40 min in some studies) typically represents the total session time commitment, including warm-up, the core SIT protocol (sprints and recovery), and cool-down periods. This clarifies the potential discrepancy with the 10 min–20 min timeframe mentioned in the introduction, which often refers more narrowly to the core protocol itself or the total exercise volume time and may not include the full duration of auxiliary activities. The predominant exercise modalities were cycling and running, with only one study utilizing walking. SIT protocols featured single sprint durations ranging from 10 s to 40 s, with the number of sprints varying from 4 to 40 repetitions. Exercise intensity was prescribed as “all-out,” “maximum-effort,” or at a supramaximal level based on physiological markers such as maximal oxygen uptake (VO_2_max) or maximal aerobic speed (MAS) (e.g., 120% MAS, 170% peak power).

**TABLE 2:** Details of the exercise intervention protocols.

Study	Group	Exercise modality	Duration (wk)	Frequency (time/wk)	Session duration (min)	Intensity	No. of reps	Rep duration(s)	Work–rest ratio	Adherence (%)
Boer et al. (2014)	SIT	Cycling	15	2	40	VT -> 110% VT	20 (2 × 10)	15	1:03	>95
	MICT	Cycling, running, and stepping	15	2	40	VT -> 110% VT	-	-	-	>95
	CON	-	15	-	-	-	-	-	-	-
Chuensiri et al. (2018)	SIT	Cycling	12	3	∼21	170% peak power	8	20	01:00.5	NR
	CON	-	12	-	-	-	-	-	-	-
Lau et al. (2015)	SIT	Running	6	3	6 (work only)	120% MAS	12	15	1:01	NR
	CON	-	6	-	-	-	-	-	-	-
Lazzer et al. (2017)	SIT	Walking	3	10	37	100% VO_2_peak	6	40	01:07.5	>97
	MICT	Walking	3	10	31	70% VO_2_peak	-	-	-	>97
Martin et al. (2015)	SIT	Running	7	3	∼25	Sprints	4–6	30	1:01	>80
	CON	Standard PE	7	3	60	-	-	-	-	-
Martin-Smith et al. (2019)	SIT	Running	4	3	25–26	All-out	5–6	30	1:01	>80
	CON	Standard PE	4	3	60	-	-	-	-	-
Racil et al. (2013)	SIT	Running	12	3	∼20–25	100%–110% MAS	12–16	30	1:01	NR
	MICT	Running	12	3	∼20–25	70%–80% MAS	12–16	30	1:01	NR
	CON	-	12	-	-	-	-	-	-	-
Rosenkranz et al. (2012)	SIT	Running	8	2	∼30	100%–130% MAS	40–20	10–20	1:01	NR
	CON	-	8	-	-	-	-	-	-	-
Salus et al. (2022a)	SIT	Cycling	12	3	29–38	All-out	4–6	30	1:08	80–89
	CON	-	12	-	-	-	-	-	-	-
Salus et al., 202b	SIT	Cycling	12	3	29–38	All-out	4–6	30	1:08	80–89
	CON	-	12	-	-	-	-	-	-	-
Su et al. (2025)	SIT	Cycling	6	3	21–30	All-out (7.5% BW)	4–6	30	1:08	100
	MICT	Cycling	6	3	30–60	65% VO_2_peak	-	-	-	100
Tadiotto et al. (2023)	SIT	Cycling	12	3	35	80%–100% HRreserve	12 (3 × 4)	30	1:02	>75
	MICT	Cycling	12	3	60	35%–75% HRreserve	-	-	-	>75
	CON	-	12	-	-	-	-	-	-	-
Taylor et al. (2019)	SIT	Cycling	8	3	18	Maximum effort	4	30	1:08	75.7
	CON	-	8	-	-	-	-	-	-	-
Williams et al. (2022)	SIT	Running	2	3	8–10	Maximum effort	6–8	10	1:05	97.9
	CON	-	2	-	-	-	-	-	-	-

BW, body weight; CON, control group (no-exercise or standard physical education); HIIT, high-intensity interval training; HRmax, maximum heart rate; HRreserve, heart rate reserve; MAS, maximal aerobic speed; MICT, moderate-intensity continuous training; NR, not reported; reps, repetitions; SIT, sprint interval training; Standard PE, standard physical education; VO_2_peak/max, peak/maximal oxygen consumption; VT, ventilatory threshold; wk, week(s).

For control groups, 12 studies incorporated a non-training or standard physical education control group (CON). Furthermore, five studies included an MICT arm. MICT protocols typically consisted of 30 min–60 min of continuous aerobic exercise at an intensity of 65%–80% of the maximum heart rate or VO_2_max. Notably, three studies featured a three-arm design, comparing SIT, MICT, and CON groups simultaneously. Most studies reported high adherence rates to the interventions, generally exceeding 80%.

### 4.3 Risk of bias assessment

The results of the risk-of-bias assessment for all 14 included studies are detailed in Supplementary Figures S2, S3. Overall, no study was judged to have a “low risk of bias.” Five studies (35.7%) were assessed as having a “high risk of bias,” while the remaining nine (64.3%) were rated as having “some concerns.” The primary source of bias stemmed from the “randomization process.” The high-risk ratings were predominantly attributed to the use of non-random or cluster-randomized designs in several studies. The “some concerns” ratings were mainly due to insufficient detail regarding the randomization methodology, the inherent difficulty of blinding participants and personnel to exercise interventions, and the failure to ensure assessor blinding for outcomes such as physical fitness. Additionally, high attrition rates in some studies raised further concerns. All included studies were deemed to have a low risk of bias related to selective reporting of results.

### 4.4 Meta-analysis

#### 4.4.1 Effects of SIT versus control

**FIGURE 2:**
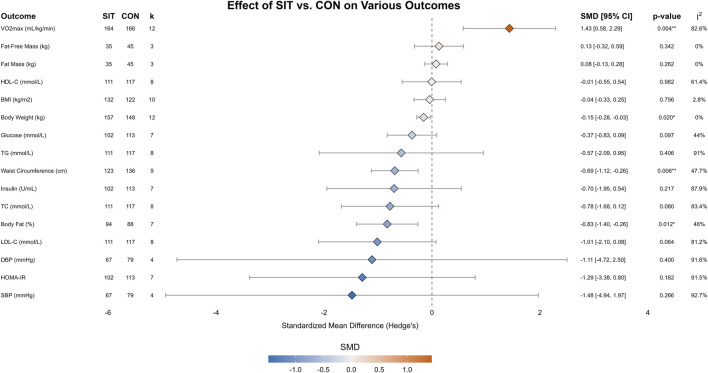
Forest plot of the meta-analysis comparing the effects of SIT versus CON on cardiometabolic and physical fitness outcomes. Diamonds represent the pooled SMD and 95% confidence intervals using a random-effects model.

A detailed summary of the meta-analysis comparing SIT to the non-exercise control group is provided in Table 3, with forest plots presented in Figure 2. Compared to the CON group, SIT induced a very large and significant improvement in cardiorespiratory fitness (VO_2_max) [SMD = 1.43, 95% CI (0.58, 2.29); *p* = 0.004; I^2^ = 82.6%]. Significant improvements were also observed in several body composition measures, including a large reduction in the body fat percentage [SMD = −0.83, 95% CI (-1.40, −0.26); *p* = 0.012; I^2^ = 46%], a moderate decrease in waist circumference [SMD = −0.69, 95% CI (-1.12, −0.26); *p* = 0.006; I^2^ = 47.7%], and a small but significant decrease in body weight [SMD = −0.15, 95% CI (-0.28, −0.03); *p* = 0.020; I^2^ = 0%]. No statistically significant effects were observed in other body composition measures or any cardiac, metabolic, or blood measures.

**TABLE 3:** Meta-analysis of the effects of SIT versus CON.

Outcome	No. of studies	Group	N	Pooled difference (95% CI)	P	Heterogeneity I^2^, *p*
BMI (kg/m^2^)	10	SIT	132	−0.04 [−0.33, 0.25]	0.756	2.8%, 0.41
		CON	122			
Body fat (%)	7	SIT	94	−0.83 [−1.40, −0.26]	0.012*	46%, 0.08
		CON	88			
Body weight (kg)	12	SIT	157	−0.15 [−0.28, −0.03]	0.020*	0%, 0.99
		CON	148			
DBP (mmHg)	4	SIT	67	−1.11 [−4.72, 2.50]	0.4	91.6%, <0.001
		CON	79			
Fat mass (kg)	3	SIT	35	0.08 [−0.13, 0.28]	0.262	0%, 0.96
		CON	45			
Fat-free mass (kg)	3	SIT	35	0.13 [−0.32, 0.59]	0.342	0%, 0.81
		CON	45			
Glucose (mmol/L)	7	SIT	102	−0.37 [−0.83, 0.09]	0.097	44%, 0.10
		CON	113			
HDL-C (mmol/L)	8	SIT	111	−0.01 [−0.55, 0.54]	0.982	61.4%, 0.01
		CON	117			
HOMA-IR	7	SIT	102	−1.29 [−3.38, 0.80]	0.182	91.5%, <0.001
		CON	113			
Insulin (U/mL)	7	SIT	102	−0.70 [−1.95, 0.54]	0.217	87.9%, <0.001
		CON	113			
LDL-C (mmol/L)	8	SIT	111	−1.01 [−2.10, 0.08]	0.064	81.2%, <0.001
		CON	117			
SBP (mmHg)	4	SIT	67	−1.48 [−4.94, 1.97]	0.266	92.7%, <0.001
		CON	79			
TC (mmol/L)	8	SIT	111	−0.78 [−1.68, 0.12]	0.08	83.4%, <0.001
		CON	117			
TG (mmol/L)	8	SIT	111	−0.57 [−2.09, 0.95]	0.406	91%, <0.001
		CON	117			
VO_2_max (mL/kg/min)	12	SIT	164	1.43 [0.58, 2.29]	0.004**	82.6%, <0.001
		CON	166			
Waist circumference (cm)	9	SIT	123	−0.69 [−1.12, −0.26]	0.006**	47.7%, 0.05
		CON	136			

Data are presented as the pooled standardized mean differences (SMD) with 95% confidence intervals (CI) calculated using a random-effects model. Abbreviations: SIT, sprint interval training; CON, control group; N, number of participants; I^2^, I-squared statistic for heterogeneity; BMI, body mass index; DBP, diastolic blood pressure; HDL-C, high-density lipoprotein cholesterol; HOMA-IR, homeostatic model assessment for insulin resistance; LDL-C, low-density lipoprotein cholesterol; SBP, systolic blood pressure; TC, total cholesterol; TG, triglycerides; VO_2_max, maximal oxygen uptake. *p < 0.05; **p < 0.01.

#### 4.4.2 Effects of SIT versus MICT

**FIGURE 3:**
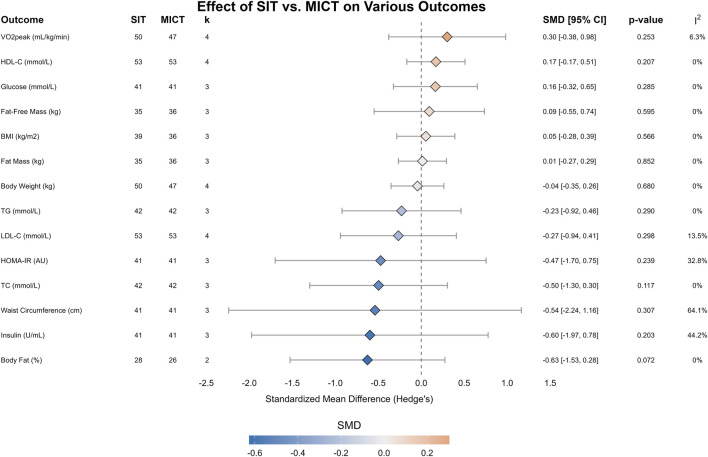
Forest plot of the meta-analysis comparing the effects of SIT versus MICT on cardiometabolic and physical fitness outcomes. Diamonds represent the pooled standardized mean difference (SMD) and 95% confidence intervals using a random-effects model.

The comparative effects of SIT and MICT are summarized in Table 4 and visualized in Figure 3. The analysis revealed no statistically significant differences between the SIT and MICT groups across all measured outcomes. For the key outcome of cardiorespiratory fitness (VO_2_max), the effects of the two interventions were comparable [SMD = 0.30, 95% CI (-0.38, 0.98); *p* = 0.253; I^2^ = 6.3%].

**TABLE 4:** Meta-analysis of the effects of SIT versus MICT.

Outcome	No. of studies	Exercise	N	Pooled difference (95% CI)	P	Heterogeneity I^2^, *p*
Body fat (%)	2	SIT	28	−0.63 [−1.53, 0.28]	0.072	0%, 0.80
		MICT	26			
Insulin (U/mL)	3	SIT	41	−0.60 [−1.97, 0.78]	0.203	44.2%, 0.17
		MICT	41			
Waist circumference (cm)	3	SIT	41	−0.54 [−2.24, 1.16]	0.307	64.1%, 0.06
		MICT	41			
TC (mmol/L)	3	SIT	42	−0.50 [−1.30, 0.30]	0.117	0%, 0.50
		MICT	42			
HOMA-IR (AU)	3	SIT	41	−0.47 [−1.70, 0.75]	0.239	32.8%, 0.23
		MICT	41			
LDL-C (mmol/L)	4	SIT	53	−0.27 [−0.94, 0.41]	0.298	13.5%, 0.32
		MICT	53			
TG (mmol/L)	3	SIT	42	−0.23 [−0.92, 0.46]	0.29	0%, 0.59
		MICT	42			
Body weight (kg)	4	SIT	50	−0.04 [−0.35, 0.26]	0.68	0%, 0.88
		MICT	47			
Fat Mass (kg)	3	SIT	35	0.01 [−0.27, 0.29]	0.852	0%, 0.93
		MICT	36			
BMI (kg/m^2^)	3	SIT	39	0.05 [−0.28, 0.39]	0.566	0%, 0.89
		MICT	36			
Fat-free Mass (kg)	3	SIT	35	0.09 [−0.55, 0.74]	0.595	0%, 0.68
		MICT	36			
Glucose (mmol/L)	3	SIT	41	0.16 [−0.32, 0.65]	0.285	0%, 0.77
		MICT	41			
HDL-C (mmol/L)	4	SIT	53	0.17 [−0.17, 0.51]	0.207	0%, 0.83
		MICT	53			
VO_2_peak (mL/kg/min)	4	SIT	50	0.30 [−0.38, 0.98]	0.253	6.3%, 0.36
		MICT	47			

Data are presented as the pooled standardized mean differences (SMD) with 95% confidence intervals (CI) calculated using a random-effects model. Abbreviations: SIT, sprint interval training; MICT, moderate-intensity continuous training; N, number of participants; I^2^, I-squared statistic for heterogeneity; BMI, body mass index; HDL-C, high-density lipoprotein cholesterol; HOMA-IR, homeostatic model assessment for insulin resistance; LDL-C, low-density lipoprotein cholesterol; TC, total cholesterol; TG, triglycerides; VO_2_peak, peak oxygen uptake.

### 4.5 Subgroup and sensitivity analysis

To investigate the potential sources of heterogeneity, we conducted subgroup analyses based on the participants’ weight status, sex, age, and the SIT exercise modality (Supplementary Table S2). These analyses indicated that the beneficial effects of SIT on the body composition were particularly pronounced in adolescents who were overweight or obese. Interestingly, regarding exercise modality, running-based SIT appeared more advantageous for improving select lipid and glucose markers, whereas cycling-based SIT was more effective in reducing body fat. However, these findings should be interpreted with caution due to the small number of studies in many subgroups.

A leave-one-out sensitivity analysis was performed for outcomes with a sufficient number of included studies (Supplementary Figures S3–S18). The results demonstrated that the sequential removal of individual studies did not substantially alter the pooled effect estimates for any outcome. This indicates that the main findings of this meta-analysis are robust and not unduly influenced by any single study.

### 4.6 Publication bias

Funnel plots were generated to assess the publication bias for outcomes with 10 or more included studies (VO_2_max, body weight, and BMI). Visual inspection of the funnel plots for these three outcomes revealed largely symmetrical distributions, suggesting no evidence of significant, systematic publication bias (Supplementary Figures S19–S31).

### 4.7 Summary of findings

The quality of evidence for all primary outcomes was assessed using the GRADE methodology (Table 5). The evidence was rated as “moderate” quality for the effects of SIT on improving cardiorespiratory fitness and reducing the body fat percentage and body weight. The evidence for the effect of SIT on reducing waist circumference was rated as “low” quality. For all other metabolic outcomes (e.g., blood lipids, glucose, and blood pressure), the evidence was graded as “low” or “very low” quality.

**TABLE 5:** GRADE summary of the findings.

Outcome	Studies(k)/ Participants(N)	Effect estimate [SMD (95% CI)]	Certainty of evidence (GRADE)	Justification for the rating
VO_2_max (mL/kg/min)	12/330	1.43 [0.58, 2.29] (large increase)	Moderate	Downgraded once for risk of bias^a^ and once for inconsistency^b^; upgraded once for large magnitude of effect^c^
Body fat (%)	7/152	−0.83 [−1.48, −0.20] (large decrease)	Moderate	Downgraded once for risk of bias^a^ and once for inconsistency^b^; upgraded once for large magnitude of effect^c^
Body weight (kg)	12/305	−0.15 [−0.28, −0.03] (small decrease)	Moderate	Downgraded once for risk of bias^a^
Waist circumference (cm)	9/259	−0.69 [−1.12, −0.26] (moderate decrease)	Low	Downgraded once for risk of bias^a^ and once for inconsistency^b^
BMI (kg/m^2^)	10/254	−0.04 [−0.33, 0.25] (no significant difference)	Low	Downgraded once for risk of bias^a^ and once for imprecision^d^
Fat mass (kg)	3/90	0.08 [−0.13, 0.28] (no significant difference)	Low	Downgraded once for risk of bias^a^ and once for imprecision^d^
Fat-free mass (kg)	3/90	0.13 [−0.32, 0.58] (no significant difference)	Low	Downgraded once for risk of bias^a^ and once for imprecision^d^
Glucose (mmol/L)	7/215	−0.37 [−0.83, 0.09] (no significant difference)	Low	Downgraded once for risk of bias^a^ and once for imprecision^d^
LDL-C (mmol/L)	8/228	−1.01 [−2.10, 0.08] (no significant difference)	Very low	Downgraded once for risk of bias^a^, once for inconsistency^b^, and once for imprecision^d^
HDL-C (mmol/L)	8/228	−0.01 [−0.55, 0.54] (no significant difference)	Very low	Downgraded once for risk of bias^a^, once for inconsistency^b^, and once for imprecision^d^
TC (mmol/L)	8/228	−0.78 [−1.68, 0.12] (no significant difference)	Very low	Downgraded once for risk of bias^a^, once for inconsistency^b^, and once for imprecision^d^
TG (mmol/L)	8/228	−0.57 [−2.09, 0.93] (no significant difference)	Very low	Downgraded once for risk of bias^a^, once for inconsistency^b^, and once for imprecision^d^
Insulin (U/mL)	7/215	−0.70 [−1.95, 0.54] (no significant difference)	Very low	Downgraded once for risk of bias^a^, once for inconsistency^b^, and once for imprecision^d^
HOMA-IR	7/215	−1.29 [−3.38, 0.80] (no significant difference)	Very low	Downgraded once for risk of bias^a^, once for inconsistency^b^, and once for imprecision^d^
SBP (mmHg)	4/146	−1.48 [−4.94, 1.97] (no significant difference)	Very low	Downgraded once for risk of bias^a^, once for inconsistency^b^, and once for imprecision^d^
DBP (mmHg)	4/146	−1.11 [−4.72, 2.00] (no significant difference)	Very low	Downgraded once for risk of bias^a^, once for inconsistency^b^, and once for imprecision^d^

^a^ Risk of bias: downgraded due to serious risk of bias as the body of evidence includes studies with high or some concerns for bias.

^b^ Inconsistency: downgraded due to serious inconsistency as heterogeneity was high (I2 > 75%) and unexplained.

^c^ Large effect: upgraded because the magnitude of the effect was large (SMD >0.8) and the confidence interval was precise.

^d^ Imprecision: downgraded due to serious imprecision as the 95% confidence interval was wide and crossed the line of no effect.

## 5 Discussion

**FIGURE 4:**
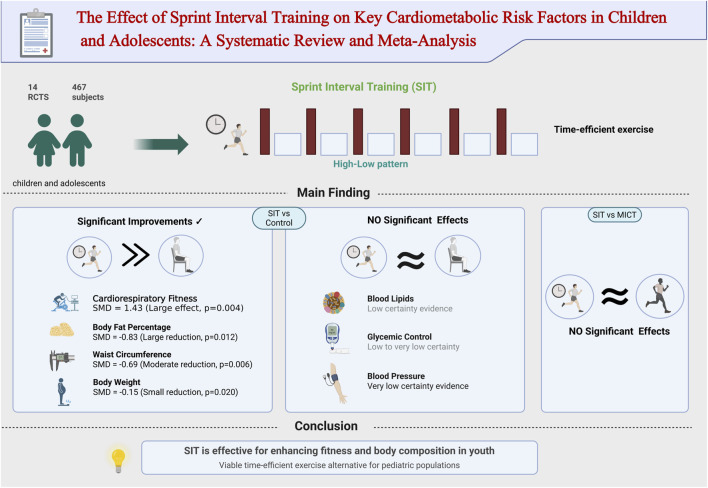
Graphical abstract of the systematic review and meta-analysis. RCTs, randomized controlled trials; SIT, sprint interval training; MICT, moderate-intensity continuous training; control, non-exercising control group; SMD, standardized mean difference.

This systematic review and meta-analysis aimed to evaluate the impact of SIT on key cardiometabolic risk factors in children and adolescents. The core findings of this meta-analysis are summarized in Figure 4. The primary finding is that compared to non-training control groups, SIT represents an efficacious strategy for improving cardiorespiratory fitness, reducing body fat percentage, and decreasing waist circumference and body weight in this population. However, when contrasted with traditional MICT, SIT demonstrated no superiority in improving any cardiometabolic parameter, suggesting that both training modalities can elicit comparable health benefits. Despite its effectiveness in enhancing cardiorespiratory function and body composition, we found no evidence that SIT significantly impacts metabolic markers such as blood lipids, glycemic control, or blood pressure.

The core finding of this study—that SIT effectively enhances cardiorespiratory fitness (VO_2_max) in children and adolescents—is highly consistent with the extensive body of research and previous meta-analyses conducted in adult populations (Gibala et al., 2012; Sloth et al., 2013). Research in adults has robustly established SIT as a time-efficient strategy for augmenting VO_2_max, with effects that are at least equivalent, if not superior, to those of MICT. Our meta-analysis is the first to quantitatively confirm that this key benefit extends to pediatric populations and that the effects are comparable between SIT and MICT. This provides a flexible and potent alternative for physical education programs and home-based exercise. A critical distinction, however, lies in the improvement of metabolic markers. Numerous studies in adults, particularly among individuals who are overweight/obese or suffer from metabolic syndrome, have reported significant improvements in insulin sensitivity and lipid profiles following SIT (Madjd et al., 2015). In contrast, our analysis did not reveal these metabolic benefits in children and adolescents. This discrepancy may be attributable to the unique physiological characteristics of the youth, who often exhibit higher baseline insulin sensitivity and healthier lipid profiles (Matthews and Pantesco, 2016), thus leaving less physiological room for significant exercise-induced improvements (the “ceiling effect”). Furthermore, the generally short duration of the interventions included in this review may have been insufficient to elicit significant alterations in blood biochemical markers.

### 5.1 Potential mechanisms

The mechanisms underlying the pronounced enhancement of cardiorespiratory fitness by SIT have been extensively investigated. This effect is largely attributed to the potent activation of peroxisome proliferator-activated receptor-gamma coactivator-1-alpha (PGC-1α) in skeletal muscle, a master regulator of mitochondrial biogenesis, which subsequently enhances muscular oxidative capacity (MacInnis and Gibala, 2017). Additionally, SIT can induce significant cardiovascular adaptations, including improvements in cardiac output and muscular oxygen extraction (Gillen et al., 2016; MacInnis and Gibala, 2017). Regarding the improvements in body composition, the high-intensity nature of SIT leads to a substantial increase in excess post-exercise oxygen consumption (EPOC), thus elevating energy expenditure for up to 24 h post-exercise (LaForgia et al., 2006). Concurrently, high-intensity exercise stimulates the secretion of hormones such as catecholamines, which promote lipolysis and fat oxidation (Jeukendrup et al., 1998; Horowitz and Klein, 2000). The lack of significant effects on metabolic markers in our study, beyond the aforementioned ceiling effect, may also be related to the complex hormonal milieu of puberty. The endogenous hormonal fluctuations characteristic of this developmental stage might mask the independent effects of the exercise intervention (Bond et al., 2017).

### 5.2 Interpretation of subgroup analyses

Our subgroup analyses offer a more nuanced perspective on the application of SIT across different subpopulations. An important observation is that the benefits of SIT on body composition (i.e., waist circumference and body fat percentage) were predominantly observed in adolescents who were overweight or obese. This is an expected finding as this group has a greater potential for body fat reduction, and it highlights the clinical utility of SIT as a weight management strategy (Dias et al., 2018). Intriguingly, with respect to exercise modality, running-based SIT appeared more advantageous for improving select lipid and glucose markers, whereas cycling was more effective for reducing body fat. This may be related to running being a weight-bearing activity that engages a larger muscle mass, potentially eliciting a more pronounced systemic response (Bijker et al., 2002; Nieman et al., 2014). However, this observation requires confirmation through head-to-head comparative studies. Similarly, subgroup analyses by sex and age suggested potential differences, but these results should be considered exploratory and hypothesis-generating for future research due to the limited number of studies within each subgroup.

### 5.3 Strengths and limitations

This review possesses several strengths. First, we conducted a comprehensive and systematic search of six major databases and adhered to the PRISMA statement, ensuring transparency and reproducibility. Second, by including both RCTs and non-RCTs and applying rigorous tools for risk-of-bias assessment (RoB 2) and evidence grading (GRADE), we have provided a thorough evaluation of the quality of the existing evidence. Finally, through diverse subgroup and sensitivity analyses, we explored the sources of heterogeneity and confirmed the robustness of our findings.

Nevertheless, some limitations must be acknowledged. First, the overall quality of the included studies is suboptimal; no study was rated as having a low risk of bias, primarily due to the inherent challenges of blinding in exercise interventions and deficiencies in the randomization process of some studies. Second, significant statistical heterogeneity was present for certain outcomes (e.g., VO_2_max), and although partially explained by our subgroup analyses, some sources remain unidentified. Third, our analysis combined “non-training” control groups with “standard physical education” control groups into a single ‘CON’ category. This represents a notable limitation. Participants in standard physical education groups receive some level of structured physical activity, however minimal, which is distinct from the baseline activity level of a “non-training” or “sedentary” control group. Pooling these groups may introduce heterogeneity and could potentially underestimate the true effect size of SIT when compared with a genuinely inactive control. A separate subgroup analysis for these two control types was not feasible due to the limited number of primary studies. Future research should clearly differentiate these control groups, and subsequent meta-analyses with sufficient data should analyze them separately to provide a more precise estimate of SIT’s intervention effects. Fourth, our literature search was confined to publications in English, and we did not extend our search to non-English databases such as the China National Knowledge Infrastructure (CNKI), VIP, and Wanfang. This restriction introduces a potential for language bias and may have led to the omission of relevant studies published in other languages. Fifth, limitations also stem from the methodologies of the primary studies. A major concern is the general lack of rigorous dietary control in most studies. As diet is a critical factor influencing cardiometabolic outcomes, unmonitored changes in dietary habits could have served as a significant confounding variable. Furthermore, limitations in the data reported by the primary studies precluded more in-depth analyses of other potential confounding factors, such as the pubertal stage. The substantial heterogeneity in SIT protocols across studies (e.g., work-to-rest ratios and sprint intensity details) also precluded a formal dose–response analysis. Finally, it is important to acknowledge the scope of our review. The term ‘cardiometabolic health’ encompasses a wide array of physiological indicators. Our analysis was limited to a selection of commonly measured risk factors, namely, cardiorespiratory fitness, body composition, blood lipids, glycemic control parameters, and blood pressure. We did not synthesize data on other important markers such as endothelial function, arterial stiffness, or systemic inflammation (e.g., C-reactive protein). Therefore, our conclusions should be interpreted within the context of these specific outcomes, and the effect of SIT on the broader spectrum of cardiometabolic health in the youth remains to be fully elucidated.

### 5.4 Implications for practice and future research

Based on our findings, several practical implications can be drawn. For children and adolescents seeking to enhance cardiorespiratory fitness and improve body composition, SIT is a valid and time-efficient training option. Given its comparable efficacy to MICT, clinicians, physical education teachers, and coaches can choose between the two modalities based on individual preferences, time constraints, and equipment availability. SIT may be particularly appealing for time-poor adolescents.

Future research should prioritize conducting high-quality, large-scale randomized controlled trials with detailed reporting according to the CONSORT statement. Particular attention should be paid to the following areas: 1. directly comparing different SIT protocols (e.g., running vs. cycling; varying work-to-rest ratios) on multifaceted health outcomes; 2. implementing longer-term interventions (e.g., >12 weeks) to assess the chronic effects on metabolic markers; 3. rigorously controlling for and reporting on confounding variables such as the diet, habitual physical activity, and pubertal status; and 4. further examining the therapeutic potential of SIT in specific pediatric clinical populations diagnosed with metabolic abnormalities, such as insulin resistance or dyslipidemia.

## 6 Conclusion

In conclusion, this systematic review and meta-analysis demonstrates that SIT is an efficacious strategy for enhancing cardiorespiratory fitness and improving body composition—particularly by reducing the body fat percentage and waist circumference—in children and adolescents, with effects comparable to those of traditional MICT. However, the current evidence does not support a significant role for SIT in improving other cardiometabolic markers, such as blood lipids, glycemic control, or blood pressure, in this population. Although SIT can serve as a time-efficient exercise alternative, its comprehensive role in mitigating the overall cardiometabolic risk in youth requires further elucidation through higher-quality research.

## Data Availability

The original contributions presented in the study are included in the article/Supplementary Material, further inquiries can be directed to the corresponding authors.
