# Supplementary material for: The effect of sprint interval training on key cardiometabolic risk factors in children and adolescents: a systematic review and meta-analysis
Source: Front Physiol. 2025 Dec 18;16:1694357. doi: 10.3389/fphys.2025.1694357 (PMC12756142; doi:10.3389/fphys.2025.1694357)
Supplement: Supplementary file 1 [file DataSheet1.pdf]

# *Supplementary Material*

Table S1. Search Strategy.

Table S2. Summary of subgroup analyses for outcomes.

Figure S1. Risk of bias summary.

Figure S2. Risk of bias traffic light plot.

Figures S3-S18. Leave-one-out sensitivity analyses.

Figures S19-S21. Funnel plots for the assessment of publication bias.

**Table S1. Search Strategy.**

Pubmed

| | |
|---|---|
| #1 | "sprint interval training"[Title/Abstract] OR "sprint interval exercise"[Title/Abstract] OR "sprint intermittent training"[Title/Abstract] OR "sprint training"[Title/Abstract] OR "sprint-interval training"[Title/Abstract] OR "High-Intensity Interval Training"[Mesh] OR "High intensity intermittent training"[Title/Abstract] OR "High-intensity intermittent training"[Title/Abstract] OR "Interval training"[Title/Abstract] OR "interval exercise"[Title/Abstract] OR "HIIT"[Title/Abstract] OR "high intensity exercise"[Title/Abstract] OR "high intensity aerobic interval training"[Title/Abstract] OR "all-out exercise training"[Title/Abstract] OR "all-out training"[Title/Abstract] OR "all-out interval training"[Title/Abstract] OR "Supramaximal interval training"[Title/Abstract] OR "Wingate training"[Title/Abstract] OR "maximal intensity"[Title/Abstract] |
| #2 | "Child"[MeSH Terms] OR "Adolescent"[MeSH Terms] OR "Pediatrics"[MeSH Terms] OR child*[Title/Abstract] OR kid[Title/Abstract] OR kids[Title/Abstract] OR teen*[Title/Abstract] OR youth[Title/Abstract] OR youths[Title/Abstract] OR adolescent*[Title/Abstract] OR boy[Title/Abstract] OR boys[Title/Abstract] OR girl[Title/Abstract] OR girls[Title/Abstract] OR pediatric*[Title/Abstract] OR paediatric*[Title/Abstract] OR prepubertal[Title/Abstract] OR pubertal[Title/Abstract] |
| #3 | "Cardiovascular Diseases"[MeSH Terms] OR "Metabolic Syndrome"[MeSH Terms] OR "Insulin Resistance"[MeSH Terms] OR "Lipids"[MeSH Terms] OR "Blood Glucose"[MeSH Terms] OR "Oxygen Consumption"[MeSH Terms] OR "Blood Pressure"[MeSH Terms] OR cardiometabolic[Title/Abstract] OR "cardio-metabolic"[Title/Abstract] OR metabolic[Title/Abstract] OR "insulin sensitivity"[Title/Abstract] OR "insulin resistance"[Title/Abstract] OR HOMA-IR[Title/Abstract] OR glucose[Title/Abstract] OR lipid*[Title/Abstract] OR cholesterol[Title/Abstract] OR triglyceride*[Title/Abstract] OR "blood pressure"[Title/Abstract] OR "body composition"[Title/Abstract] OR "body fat"[Title/Abstract] OR BMI[Title/Abstract] OR "waist circumference"[Title/Abstract] OR "VO2max"[Title/Abstract] OR "VO2peak"[Title/Abstract] OR "cardiorespiratory fitness"[Title/Abstract] |
| #4 | #1 AND #2 AND #3 |

Web of Science

| | |
|---|---|
| #1 | TS=("sprint interval training" OR "sprint interval exercise" OR "Sprint intermittent training" OR "sprint training" OR "sprint-interval training" OR "High-Intensity Interval Training" OR "High intensity intermittent training" OR "High-intensity intermittent training" OR "Interval training" OR "interval exercise" OR "HIIT" OR "high intensity exercise" OR "high intensity aerobic interval training" OR "all-out exercise training" OR "all-out training" OR "all-out interval training" OR "Supramaximal interval training" OR "Wingate training" OR "maximal intensity") |
| #2 | TS=(child* OR kid OR kids OR teen* OR youth OR youths OR adolescent* OR boy OR boys OR girl OR girls OR pediatric* OR paediatric* OR |

| | |
|---|---|
| | prepubertal OR pubertal) |
| #3 | TS=(cardiometabolic OR "cardio-metabolic" OR metabolic OR "insulin sensitivity" OR "insulin resistance" OR HOMA-IR OR glucose OR lipid* OR cholesterol OR triglyceride* OR "blood pressure" OR "body composition" OR "body fat" OR BMI OR "waist circumference" OR "VO2max" OR "VO2peak" OR "cardiorespiratory fitness") |
| #4 | #1 AND #2 AND #3 |

SPORTDiscus

| | |
|---|---|
| S1 | TI "sprint interval training" OR AB "sprint interval training" OR TI "sprint interval exercise" OR AB "sprint interval exercise" OR TI "supramaximal interval training" OR AB "supramaximal interval training" OR TI "supramaximal interval exercise" OR AB "supramaximal interval exercise" OR TI "all-out interval training" OR AB "all-out interval training" OR TI "repeated sprint training" OR AB "repeated sprint training" OR TI "repeated sprint exercise" OR AB "repeated sprint exercise" OR TI "high-intensity interval training" OR AB "high-intensity interval training" OR TI HIIT OR AB HIIT) OR (DE "HIGH intensity interval training") ) AND (DE "EXERCISE" OR DE "SPORTS" OR DE "PHYSICAL activity") |
| S2 | TI child* OR AB child* OR TI kid OR AB kid OR TI kids OR AB kids OR TI teen* OR AB teen* OR TI youth OR AB youth OR TI youths OR AB youths OR TI adolescent* OR AB adolescent* OR TI boy OR AB boy OR TI boys OR AB boys OR TI girl OR AB girl OR TI girls OR AB girls OR TI pediatric* OR AB pediatric* OR TI paediatric* OR AB paediatric* OR TI prepubertal OR AB prepubertal OR TI pubertal OR AB pubertal) OR (DE "CHILDREN" OR DE "ADOLESCENTS" |
| S3 | TI cardiometabolic OR AB cardiometabolic OR TI "cardio-metabolic" OR AB "cardio-metabolic" OR TI metabolic OR AB metabolic OR TI "insulin sensitivity" OR AB "insulin sensitivity" OR TI "insulin resistance" OR AB "insulin resistance" OR TI HOMA-IR OR AB HOMA-IR OR TI glucose OR AB glucose OR TI lipid* OR AB lipid* OR TI cholesterol OR AB cholesterol OR TI triglyceride* OR AB triglyceride* OR TI "blood pressure" OR AB "blood pressure" OR TI "body composition" OR AB "body composition" OR TI "body fat" OR AB "body fat" OR TI BMI OR AB BMI OR TI "waist circumference" OR AB "waist circumference" OR TI "VO2max" OR AB "VO2max" OR TI "VO2peak" OR AB "VO2peak" OR TI "cardiorespiratory fitness" OR AB "cardiorespiratory fitness") OR (DE "CARDIOVASCULAR diseases" OR DE "METABOLIC syndrome" OR DE "INSULIN resistance" OR DE "LIPIDS" OR DE "BLOOD sugar" OR DE "OXYGEN consumption" OR DE "BLOOD pressure" |
| S4 | S1 AND S2 AND S3 |

Cochrane Library

| | |
|---|---|
| #1 | (sprint interval training):ti,ab,kw OR (sprint interval exercise):ti,ab,kw OR (supramaximal interval training):ti,ab,kw OR (supramaximal interval exercise):ti,ab,kw OR (all-out interval training):ti,ab,kw OR (repeated sprint training):ti,ab,kw OR (repeated sprint exercise):ti,ab,kw OR (high-intensity interval training):ti,ab,kw OR (HIIT):ti,ab,kw |

| | |
|---|---|
| #2 | [mh Exercise] OR [mh "Exercise Therapy"] OR [mh "Physical Education and Training"] OR [mh Sports] |
| #3 | #1 AND #2 |
| #4 | [mh Child] OR [mh Adolescent] OR [mh Pediatrics] |
| #5 | (child*):ti,ab,kw OR (kid):ti,ab,kw OR (kids):ti,ab,kw OR (teen*):ti,ab,kw OR (youth):ti,ab,kw OR (youths):ti,ab,kw OR (adolescent*):ti,ab,kw OR (boy):ti,ab,kw OR (boys):ti,ab,kw OR (girl):ti,ab,kw OR (girls):ti,ab,kw OR (pediatric*):ti,ab,kw OR (paediatric*):ti,ab,kw OR (prepubertal):ti,ab,kw OR (pubertal):ti,ab,kw |
| #6 | #4 OR #5 |
| #7 | [mh "Cardiovascular Diseases"] OR [mh "Metabolic Syndrome"] OR [mh "Insulin Resistance"] OR [mh Lipids] OR [mh "Blood Glucose"] OR [mh "Oxygen Consumption"] OR [mh "Blood Pressure"] |
| #8 | (cardiometabolic):ti,ab,kw OR ("cardio-metabolic"):ti,ab,kw OR (metabolic):ti,ab,kw OR ("insulin sensitivity"):ti,ab,kw OR ("insulin resistance"):ti,ab,kw OR (HOMA-IR):ti,ab,kw OR (glucose):ti,ab,kw OR (lipid*):ti,ab,kw OR (cholesterol):ti,ab,kw OR (triglyceride*):ti,ab,kw OR ("blood pressure"):ti,ab,kw OR ("body composition"):ti,ab,kw OR ("body fat"):ti,ab,kw OR (BMI):ti,ab,kw OR ("waist circumference"):ti,ab,kw OR ("VO2max"):ti,ab,kw OR ("VO2peak"):ti,ab,kw OR ("cardiorespiratory fitness"):ti,ab,kw |
| #9 | #7 OR #8 |
| #10 | #3 AND #6 AND #9 |

Embase

| | |
|---|---|
| #1 | 'sprint interval training':ab,ti OR 'sprint interval exercise':ab,ti OR 'supramaximal interval training':ab,ti OR 'supramaximal interval exercise':ab,ti OR 'all-out interval training':ab,ti OR 'repeated sprint training':ab,ti OR 'repeated sprint exercise':ab,ti OR 'high intensity interval training'/exp OR 'high-intensity interval training':ab,ti OR 'hiit':ab,ti) AND ('exercise'/exp OR 'kinesiotherapy'/exp OR 'sport'/exp |
| #2 | 'child'/exp OR 'adolescent'/exp OR 'pediatrics'/exp OR child*:ab,ti OR kid:ab,ti OR kids:ab,ti OR teen*:ab,ti OR youth:ab,ti OR youths:ab,ti OR adolescent*:ab,ti OR boy:ab,ti OR boys:ab,ti OR girl:ab,ti OR girls:ab,ti OR pediatric*:ab,ti OR paediatric*:ab,ti OR prepubertal:ab,ti OR pubertal:ab,ti |
| #3 | 'cardiometabolic risk'/exp OR 'cardiovascular disease'/exp OR 'metabolic syndrome'/exp OR 'insulin resistance'/exp OR 'lipid'/exp OR 'blood glucose'/exp OR 'oxygen consumption'/exp OR 'blood pressure'/exp OR cardiometabolic:ab,ti OR 'cardio-metabolic':ab,ti OR metabolic:ab,ti OR 'insulin sensitivity':ab,ti OR 'insulin resistance':ab,ti OR 'homa-ir':ab,ti OR glucose:ab,ti OR lipid*:ab,ti OR cholesterol:ab,ti OR triglyceride*:ab,ti OR 'blood pressure':ab,ti OR 'body composition':ab,ti OR 'body fat':ab,ti OR bmi:ab,ti OR 'waist circumference':ab,ti OR 'vo2max':ab,ti OR 'vo2peak':ab,ti OR 'cardiorespiratory fitness':ab,ti |
| #4 | #1 AND #2 AND #3 |

Scopus

| | |
|---|---|
| | TITLE-ABS-KEY("sprint interval training" OR "sprint interval exercise" OR "supramaximal interval training" OR "supramaximal interval exercise" OR "all-out interval training" OR "repeated sprint training" OR "repeated sprint exercise" OR "high-intensity interval training" OR HIIT) AND TITLE-ABS-KEY(exercise OR sport OR "physical activity")) |
| AND | |
| | (TITLE-ABS-KEY(child* OR kid OR kids OR teen* OR youth OR youths OR adolescent* OR boy OR boys OR girl OR girls OR pediatric* OR paediatric* OR prepubertal OR pubertal)) |
| AND | |
| | (TITLE-ABS-KEY(cardiometabolic OR "cardio-metabolic" OR metabolic OR "insulin sensitivity" OR "insulin resistance" OR "HOMA-IR" OR glucose OR lipid* OR cholesterol OR triglyceride* OR "blood pressure" OR "body composition" OR "body fat" OR BMI OR "waist circumference" OR "VO2max" OR "VO2peak" OR "cardiorespiratory fitness")) |

Table S2. Summary of subgroup analyses for outcomes.

| Outcomes | Subgroups | No.Studies | N | SMD(95%CI) | P | $I^2$ | P-Heterogeneity |
|---|---|---|---|---|---|---|---|
| Body Weight | Sex | | | | | | |
| | Mix | 5 | 141 | -0.15 (-0.48, 0.18) | > 0.05 | 0.00% | > 0.05 |
| | Male | 5 | 125 | -0.10 (-0.45, 0.26) | > 0.05 | 0.00% | > 0.05 |
| | Female | 2 | 39 | -0.35 (-0.99, 0.28) | > 0.05 | 0.00% | > 0.05 |
| | Weight Status | | | | | | |
| | Overweight/Obese | 7 | 182 | -0.17 (-0.46, 0.13) | > 0.05 | 0.00% | > 0.05 |
| | Normal Weight | 5 | 123 | -0.14 (-0.49, 0.22) | > 0.05 | 0.00% | > 0.05 |
| | Age | | | | | | |
| | Adolescents (>=13y) | 8 | 220 | -0.18 (-0.44, 0.09) | > 0.05 | 0.00% | > 0.05 |
| | Children (<13y) | 4 | 85 | -0.09 (-0.52, 0.33) | > 0.05 | 0.00% | > 0.05 |
| | Modality | | | | | | |
| | Cycling | 6 | 167 | -0.14 (-0.44, 0.17) | > 0.05 | 0.00% | > 0.05 |
| | Running | 6 | 138 | -0.18 (-0.51, 0.16) | > 0.05 | 0.00% | > 0.05 |
| DBP | Sex | | | | | | |
| | Mix | 3 | 120 | -0.08 (-0.74, 0.57) | > 0.05 | 65.84% | > 0.05 |
| | Male | 1 | 26 | -4.61 (-6.17, -3.05) | < 0.001*** | N/A | N/A |
| | Weight Status | | | | | | |
| | Overweight/Obese | 3 | 94 | -1.38 (-4.46, 1.70) | > 0.05 | 94.39% | < 0.001 |
| | Normal Weight | 1 | 52 | -0.42 (-0.98, 0.13) | > 0.05 | N/A | N/A |
| | Age | | | | | | |
| | Adolescents (>=13y) | 3 | 120 | -0.08 (-0.74, 0.57) | > 0.05 | 65.84% | > 0.05 |

| Outcomes | Subgroups | No.Studies | N | SMD(95%CI) | P | $I^2$ | P-Heterogeneity |
|---|---|---|---|---|---|---|---|
| | Children (<13y) | 1 | 26 | -4.61 (-6.17, -3.05) | < 0.001*** | N/A | N/A |
| | Modality | | | | | | |
| | Cycling | 3 | 94 | -1.38 (-4.46, 1.70) | > 0.05 | 94.39% | < 0.001 |
| | Running | 1 | 52 | -0.42 (-0.98, 0.13) | > 0.05 | N/A | N/A |
| Glucose | Sex | | | | | | |
| | Mix | 4 | 148 | -0.46 (-1.07, 0.15) | > 0.05 | 68.66% | 0.023 |
| | Female | 2 | 39 | -0.09 (-0.72, 0.54) | > 0.05 | 0.00% | > 0.05 |
| | Male | 1 | 28 | -0.42 (-1.17, 0.33) | > 0.05 | N/A | N/A |
| | Weight Status | | | | | | |
| | Overweight/Obese | 4 | 119 | -0.07 (-0.44, 0.29) | > 0.05 | 0.00% | > 0.05 |
| | Normal Weight | 3 | 96 | -0.77 (-1.32, -0.22) | 0.006** | 39.94% | > 0.05 |
| | Age | | | | | | |
| | Adolescents (>=13y) | 6 | 199 | -0.38 (-0.81, 0.04) | > 0.05 | 52.82% | > 0.05 |
| | Children (<13y) | 1 | 16 | -0.22 (-1.20, 0.77) | > 0.05 | N/A | N/A |
| | Modality | | | | | | |
| | Cycling | 3 | 96 | -0.09 (-0.50, 0.32) | > 0.05 | 0.00% | > 0.05 |
| | Running | 4 | 119 | -0.58 (-1.13, -0.03) | 0.040* | 51.13% | > 0.05 |
| HDL-C | Sex | | | | | | |
| | Mix | 4 | 127 | 0.22 (-0.26, 0.71) | > 0.05 | 42.15% | > 0.05 |
| | Male | 3 | 78 | -0.55 (-1.25, 0.15) | > 0.05 | 56.15% | > 0.05 |
| | Female | 1 | 23 | 0.62 (-0.22, 1.46) | > 0.05 | N/A | N/A |
| | Weight Status | | | | | | |

| Outcomes | Subgroups | No.Studies | N | SMD(95%CI) | P | $I^2$ | P-Heterogeneity |
|---|---|---|---|---|---|---|---|
| | Overweight/Obese | 5 | 145 | -0.14 (-0.85, 0.57) | > 0.05 | 75.59% | 0.003 |
| | Normal Weight | 3 | 83 | 0.18 (-0.25, 0.61) | > 0.05 | 0.00% | > 0.05 |
| | Age | | | | | | |
| | Adolescents (>=13y) | 6 | 186 | 0.09 (-0.32, 0.51) | > 0.05 | 47.77% | > 0.05 |
| | Children (<13y) | 2 | 42 | -0.34 (-2.00, 1.32) | > 0.05 | 84.32% | 0.012 |
| | Modality | | | | | | |
| | Cycling | 5 | 146 | -0.23 (-0.86, 0.40) | > 0.05 | 70.32% | 0.009 |
| | Running | 3 | 82 | 0.32 (-0.12, 0.76) | > 0.05 | 0.00% | > 0.05 |
| HOMA-IR | Sex | | | | | | |
| | Mix | 4 | 148 | -2.01 (-4.62, 0.60) | > 0.05 | 93.35% | < 0.001 |
| | Female | 2 | 39 | -0.89 (-3.35, 1.57) | > 0.05 | 91.26% | < 0.001 |
| | Male | 1 | 28 | 0.63 (-0.14, 1.39) | > 0.05 | N/A | N/A |
| | Weight Status | | | | | | |
| | Overweight/Obese | 4 | 119 | -0.64 (-1.76, 0.49) | > 0.05 | 85.16% | < 0.001 |
| | Normal Weight | 3 | 96 | -2.22 (-6.08, 1.64) | > 0.05 | 95.68% | < 0.001 |
| | Age | | | | | | |
| | Adolescents (>=13y) | 6 | 199 | -1.57 (-3.40, 0.26) | > 0.05 | 92.42% | < 0.001 |
| | Children (<13y) | 1 | 16 | 0.36 (-0.63, 1.35) | > 0.05 | N/A | N/A |
| | Modality | | | | | | |
| | Cycling | 3 | 96 | -0.19 (-1.10, 0.72) | > 0.05 | 77.50% | 0.012 |
| | Running | 4 | 119 | -2.18 (-4.89, 0.52) | > 0.05 | 93.90% | < 0.001 |
| Insulin | Sex | | | | | | |

| Outcomes | Subgroups | No.Studies | N | SMD(95%CI) | P | $I^2$ | P-Heterogeneity |
|---|---|---|---|---|---|---|---|
| | Mix | 4 | 148 | -0.78 (-1.74, 0.18) | > 0.05 | 86.12% | < 0.001 |
| | Female | 2 | 39 | -1.30 (-4.65, 2.05) | > 0.05 | 94.28% | < 0.001 |
| | Male | 1 | 28 | 0.67 (-0.10, 1.43) | > 0.05 | N/A | N/A |
| | Weight Status | | | | | | |
| | Overweight/Obese | 4 | 119 | -0.89 (-2.40, 0.61) | > 0.05 | 89.56% | < 0.001 |
| | Normal Weight | 3 | 96 | -0.48 (-1.95, 0.99) | > 0.05 | 90.34% | < 0.001 |
| | Age | | | | | | |
| | Adolescents (>=13y) | 6 | 199 | -0.88 (-1.96, 0.21) | > 0.05 | 89.23% | < 0.001 |
| | Children (<13y) | 1 | 16 | 0.39 (-0.60, 1.38) | > 0.05 | N/A | N/A |
| | Modality | | | | | | |
| | Cycling | 3 | 96 | -0.25 (-1.32, 0.81) | > 0.05 | 82.95% | 0.003 |
| | Running | 4 | 119 | -1.07 (-2.67, 0.53) | > 0.05 | 91.57% | < 0.001 |
| LDL-C | Sex | | | | | | |
| | Mix | 4 | 127 | -1.02 (-1.49, -0.55) | < 0.001*** | 35.87% | > 0.05 |
| | Male | 3 | 78 | -1.24 (-3.91, 1.44) | > 0.05 | 93.06% | < 0.001 |
| | Female | 1 | 23 | -0.37 (-1.19, 0.46) | > 0.05 | N/A | N/A |
| | Weight Status | | | | | | |
| | Overweight/Obese | 5 | 145 | -1.12 (-2.44, 0.19) | > 0.05 | 84.67% | < 0.001 |
| | Normal Weight | 3 | 83 | -0.88 (-2.09, 0.34) | > 0.05 | 81.93% | 0.004 |
| | Age | | | | | | |
| | Adolescents (>=13y) | 6 | 186 | -0.50 (-0.95, -0.05) | 0.028* | 54.56% | > 0.05 |
| | Children (<13y) | 2 | 42 | -2.95 (-5.11, -0.80) | 0.007** | 80.86% | 0.022 |

| Outcomes | Subgroups | No.Studies | N | SMD(95%CI) | P | $I^2$ | P-Heterogeneity |
|---|---|---|---|---|---|---|---|
| | Modality | | | | | | |
| | Cycling | 5 | 146 | -1.00 (-2.42, 0.41) | > 0.05 | 86.98% | < 0.001 |
| | Running | 3 | 82 | -1.06 (-1.82, -0.29) | 0.007** | 55.75% | > 0.05 |
| SBP | Sex | | | | | | |
| | Mix | 3 | 120 | -0.52 (-1.68, 0.64) | > 0.05 | 85.73% | < 0.001 |
| | Male | 1 | 26 | -4.63 (-6.20, -3.06) | < 0.001*** | N/A | N/A |
| | Weight Status | | | | | | |
| | Overweight/Obese | 3 | 94 | -1.98 (-4.70, 0.74) | > 0.05 | 94.47% | < 0.001 |
| | Normal Weight | 1 | 52 | -0.11 (-0.66, 0.44) | > 0.05 | N/A | N/A |
| | Age | | | | | | |
| | Adolescents (>=13y) | 3 | 120 | -0.52 (-1.68, 0.64) | > 0.05 | 85.73% | < 0.001 |
| | Children (<13y) | 1 | 26 | -4.63 (-6.20, -3.06) | < 0.001*** | N/A | N/A |
| | Modality | | | | | | |
| | Cycling | 3 | 94 | -1.98 (-4.70, 0.74) | > 0.05 | 94.47% | < 0.001 |
| | Running | 1 | 52 | -0.11 (-0.66, 0.44) | > 0.05 | N/A | N/A |
| TC | Sex | | | | | | |
| | Mix | 4 | 127 | -1.11 (-1.69, -0.52) | < 0.001*** | 54.64% | > 0.05 |
| | Male | 3 | 78 | -0.50 (-2.50, 1.51) | > 0.05 | 92.57% | < 0.001 |
| | Female | 1 | 23 | -0.30 (-1.13, 0.52) | > 0.05 | N/A | N/A |
| | Weight Status | | | | | | |
| | Overweight/Obese | 5 | 145 | -0.60 (-1.67, 0.48) | > 0.05 | 86.37% | < 0.001 |
| | Normal Weight | 3 | 83 | -1.10 (-2.08, -0.11) | 0.029* | 76.14% | 0.015 |

| Outcomes | Subgroups | No.Studies | N | SMD(95%CI) | P | $I^2$ | P-Heterogeneity |
|---|---|---|---|---|---|---|---|
| | Age | | | | | | |
| | Adolescents (>=13y) | 6 | 186 | -0.43 (-1.16, 0.30) | > 0.05 | 81.93% | < 0.001 |
| | Children (<13y) | 2 | 42 | -2.01 (-2.95, -1.07) | < 0.001*** | 30.99% | > 0.05 |
| | Modality | | | | | | |
| | Cycling | 5 | 146 | -0.57 (-1.65, 0.52) | > 0.05 | 86.56% | < 0.001 |
| | Running | 3 | 82 | -1.15 (-2.04, -0.26) | 0.011* | 69.50% | 0.038 |
| TG | Sex | | | | | | |
| | Mix | 4 | 127 | -0.77 (-1.76, 0.22) | > 0.05 | 84.58% | < 0.001 |
| | Male | 3 | 78 | -0.44 (-4.05, 3.16) | > 0.05 | 96.22% | < 0.001 |
| | Female | 1 | 23 | -0.17 (-0.99, 0.65) | > 0.05 | N/A | N/A |
| | Weight Status | | | | | | |
| | Overweight/Obese | 5 | 145 | -0.27 (-2.21, 1.67) | > 0.05 | 92.57% | < 0.001 |
| | Normal Weight | 3 | 83 | -1.08 (-2.22, 0.07) | > 0.05 | 81.95% | 0.004 |
| | Age | | | | | | |
| | Adolescents (>=13y) | 6 | 186 | 0.10 (-0.93, 1.13) | > 0.05 | 88.77% | < 0.001 |
| | Children (<13y) | 2 | 42 | -2.77 (-4.94, -0.60) | 0.012* | 82.26% | 0.018 |
| | Modality | | | | | | |
| | Cycling | 5 | 146 | -0.23 (-2.17, 1.71) | > 0.05 | 92.53% | < 0.001 |
| | Running | 3 | 82 | -1.15 (-2.16, -0.14) | 0.026* | 76.05% | 0.015 |
| VO2max | Sex | | | | | | |
| | Mix | 5 | 166 | 1.16 (0.80, 1.52) | < 0.001*** | 0.00% | > 0.05 |
| | Male | 5 | 125 | 1.96 (0.14, 3.79) | 0.035* | 92.53% | < 0.001 |

| Outcomes | Subgroups | No.Studies | N | SMD(95%CI) | P | $I^2$ | P-Heterogeneity |
|---|---|---|---|---|---|---|---|
| | Female | 2 | 39 | 1.11 (-0.56, 2.77) | > 0.05 | 81.69% | 0.019 |
| | Weight Status | | | | | | |
| | Overweight/Obese | 6 | 155 | 2.09 (0.76, 3.43) | 0.002** | 88.47% | < 0.001 |
| | Normal Weight | 6 | 175 | 0.87 (0.34, 1.40) | 0.001** | 61.76% | 0.023 |
| | Age | | | | | | |
| | Adolescents (>=13y) | 9 | 272 | 1.26 (0.58, 1.94) | < 0.001*** | 80.19% | < 0.001 |
| | Children (<13y) | 3 | 58 | 2.11 (-0.50, 4.72) | > 0.05 | 90.97% | < 0.001 |
| | Modality | | | | | | |
| | Cycling | 6 | 167 | 1.91 (0.54, 3.28) | 0.006** | 89.23% | < 0.001 |
| | Running | 6 | 163 | 1.04 (0.39, 1.69) | 0.002** | 69.08% | 0.006 |
| Waist Circumference | Sex | | | | | | |
| | Mix | 5 | 166 | -0.54 (-0.97, -0.11) | 0.013* | 41.57% | > 0.05 |
| | Male | 2 | 54 | -1.38 (-1.99, -0.78) | < 0.001*** | 0.00% | > 0.05 |
| | Female | 2 | 39 | -0.40 (-1.10, 0.30) | > 0.05 | 14.76% | > 0.05 |
| | Weight Status | | | | | | |
| | Overweight/Obese | 5 | 145 | -1.08 (-1.45, -0.71) | < 0.001*** | 5.19% | > 0.05 |
| | Normal Weight | 4 | 114 | -0.22 (-0.59, 0.15) | > 0.05 | 0.00% | > 0.05 |
| | Age | | | | | | |
| | Adolescents (>=13y) | 6 | 201 | -0.68 (-1.08, -0.29) | < 0.001*** | 42.58% | > 0.05 |
| | Children (<13y) | 3 | 58 | -0.67 (-1.67, 0.32) | > 0.05 | 69.50% | 0.038 |
| | Modality | | | | | | |
| | Cycling | 5 | 152 | -0.95 (-1.49, -0.41) | < 0.001*** | 59.00% | 0.045 |

frontiers

| Outcomes | Subgroups | No.Studies | N | SMD(95%CI) | P | $I^2$ | P-Heterogeneity |
|---|---|---|---|---|---|---|---|
| | Running | 4 | 107 | -0.36 (-0.74, 0.03) | > 0.05 | 0.00% | > 0.05 |
| BMI | Sex | | | | | | |
| | Mix | 5 | 141 | -0.25 (-0.58, 0.08) | > 0.05 | 0.00% | > 0.05 |
| | Male | 4 | 97 | 0.28 (-0.25, 0.80) | > 0.05 | 39.77% | > 0.05 |
| | Female | 1 | 16 | 0.00 (-0.98, 0.98) | > 0.05 | N/A | N/A |
| | Weight Status | | | | | | |
| | Overweight/Obese | 5 | 131 | 0.13 (-0.32, 0.58) | > 0.05 | 40.17% | > 0.05 |
| | Normal Weight | 5 | 123 | -0.20 (-0.55, 0.16) | > 0.05 | 0.00% | > 0.05 |
| | Age | | | | | | |
| | Adolescents (>=13y) | 6 | 169 | -0.14 (-0.44, 0.16) | > 0.05 | 0.00% | > 0.05 |
| | Children (<13y) | 4 | 85 | 0.15 (-0.50, 0.80) | > 0.05 | 53.58% | > 0.05 |
| | Modality | | | | | | |
| | Cycling | 5 | 139 | 0.09 (-0.34, 0.52) | > 0.05 | 40.00% | > 0.05 |
| | Running | 5 | 115 | -0.17 (-0.54, 0.20) | > 0.05 | 0.00% | > 0.05 |
| Body Fat | Sex | | | | | | |
| | Mix | 3 | 77 | -0.45 (-0.91, 0.00) | > 0.05 | 0.00% | > 0.05 |
| | Male | 3 | 82 | -0.89 (-1.35, -0.43) | < 0.001*** | 0.00% | > 0.05 |
| | Female | 1 | 23 | -2.32 (-3.42, -1.22) | < 0.001*** | N/A | N/A |
| | Weight Status | | | | | | |
| | Overweight/Obese | 5 | 136 | -1.03 (-1.44, -0.62) | < 0.001*** | 38.73% | > 0.05 |
| | Normal Weight | 2 | 46 | -0.27 (-0.85, 0.31) | > 0.05 | 0.00% | > 0.05 |
| | Age | | | | | | |

| Outcomes | Subgroups | No.Studies | N | SMD(95%CI) | P | $I^2$ | P-Heterogeneity |
|---|---|---|---|---|---|---|---|
| | Adolescents (>=13y) | 5 | 140 | -0.98 (-1.53, -0.43) | < 0.001*** | 56.47% | > 0.05 |
| | Children (<13y) | 2 | 42 | -0.49 (-1.12, 0.13) | > 0.05 | 0.00% | > 0.05 |
| | Modality | | | | | | |
| | Cycling | 5 | 143 | -0.73 (-1.07, -0.39) | < 0.001*** | 0.00% | > 0.05 |
| | Running | 2 | 39 | -1.23 (-3.34, 0.87) | > 0.05 | 87.77% | 0.004 |
| Fat Mass | Sex | | | | | | |
| | Male | 2 | 43 | 0.06 (-0.54, 0.66) | > 0.05 | 0.00% | > 0.05 |
| | Mix | 1 | 37 | 0.09 (-0.58, 0.77) | > 0.05 | N/A | N/A |
| | Weight Status | | | | | | |
| | Overweight/Obese | 2 | 56 | 0.12 (-0.42, 0.66) | > 0.05 | 0.00% | > 0.05 |
| | Normal Weight | 1 | 24 | -0.02 (-0.82, 0.78) | > 0.05 | N/A | N/A |
| | Modality | | | | | | |
| | Running | 1 | 19 | 0.16 (-0.74, 1.07) | > 0.05 | N/A | N/A |
| | Cycling | 2 | 61 | 0.05 (-0.47, 0.56) | > 0.05 | 0.00% | > 0.05 |
| Fat-Free Mass | Sex | | | | | | |
| | Male | 2 | 43 | 0.26 (-0.34, 0.86) | > 0.05 | 0.00% | > 0.05 |
| | Mix | 1 | 37 | -0.04 (-0.71, 0.64) | > 0.05 | N/A | N/A |
| | Weight Status | | | | | | |
| | Overweight/Obese | 2 | 56 | 0.08 (-0.46, 0.62) | > 0.05 | 0.00% | > 0.05 |
| | Normal Weight | 1 | 24 | 0.25 (-0.55, 1.05) | > 0.05 | N/A | N/A |
| | Modality | | | | | | |
| | Running | 1 | 19 | 0.28 (-0.62, 1.19) | > 0.05 | N/A | N/A |

| Outcomes | Subgroups | No.Studies | N | SMD(95%CI) | P | $I^2$ | P-Heterogeneity |
|---|---|---|---|---|---|---|---|
| | Cycling | 2 | 61 | 0.08 (-0.44, 0.60) | > 0.05 | 0.00% | > 0.05 |

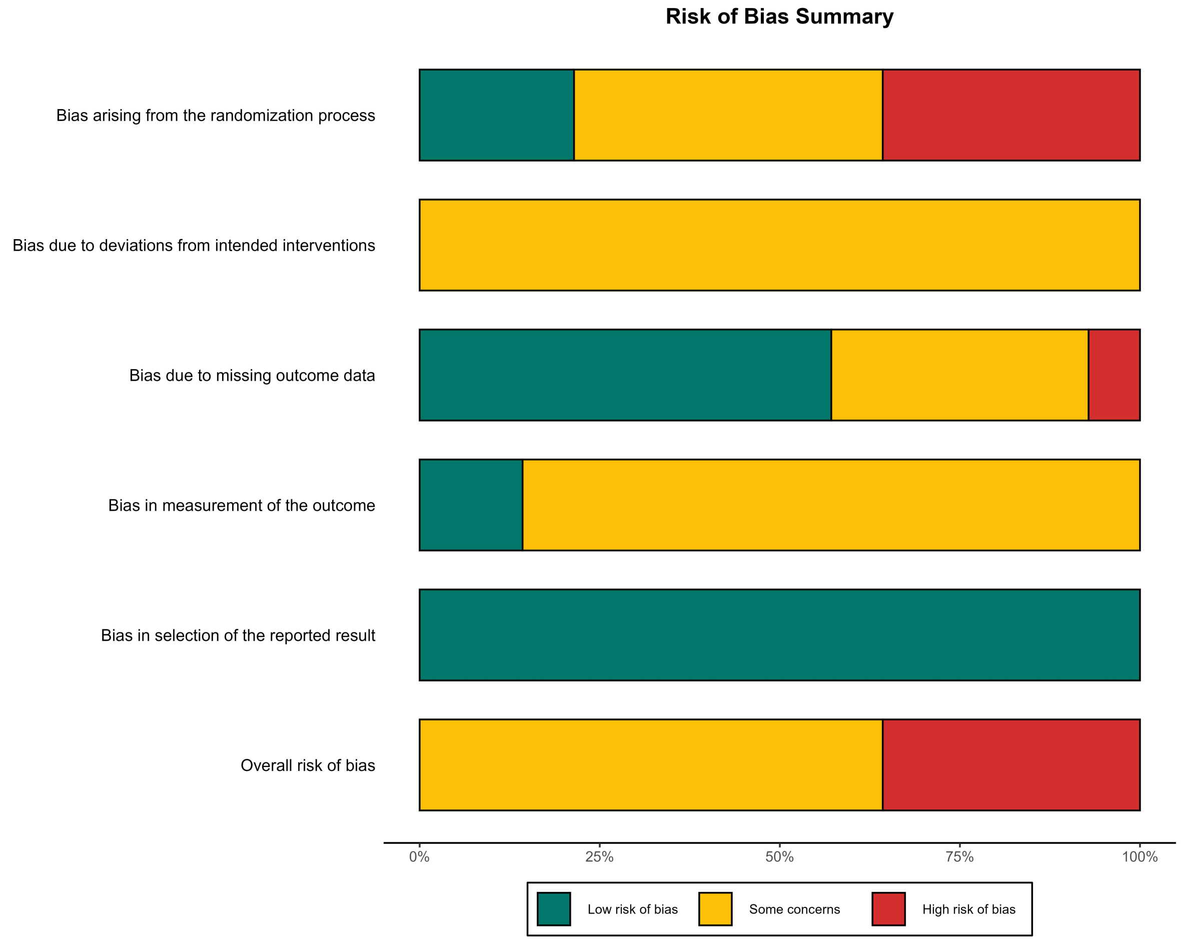


**Figure S1. Risk of bias summary.** The bar chart displays the distribution of risk of bias judgments (low risk, some concerns, high risk) for each domain of the Cochrane RoB 2 tool across all included studies.

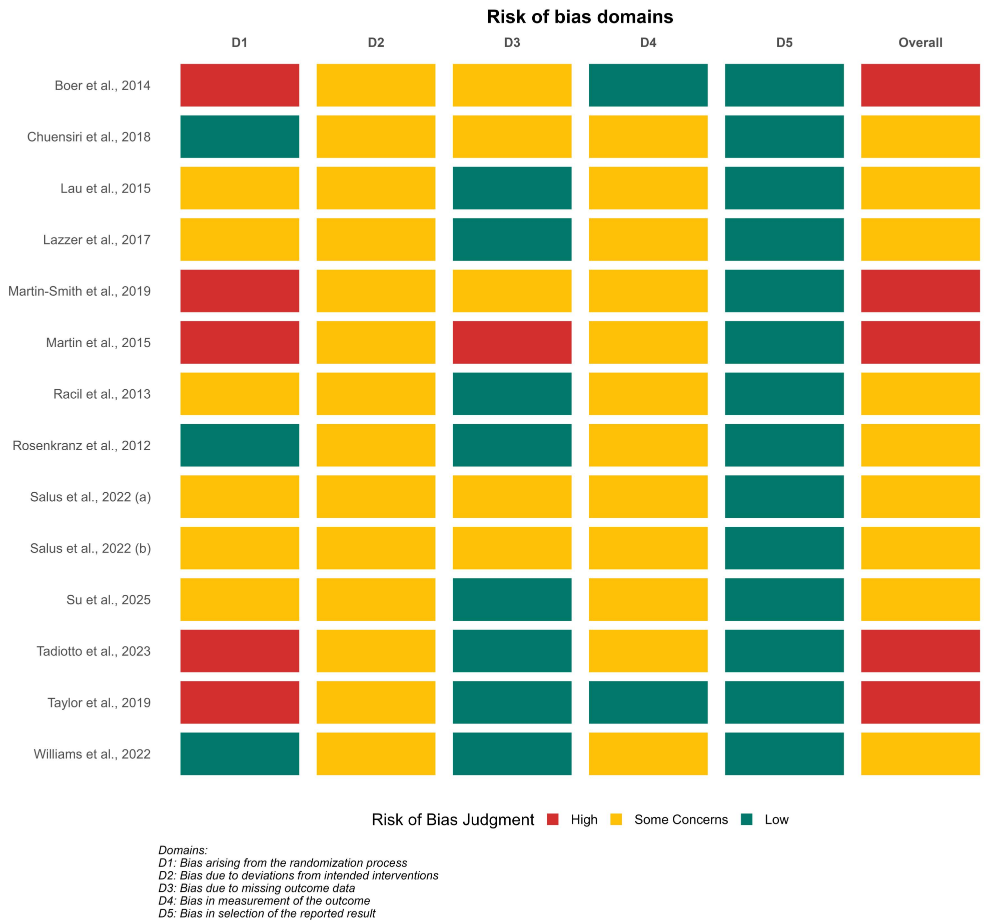


**Figure S2. Risk of bias traffic light plot.** Each row represents an individual study, and each column represents a specific risk of bias domain from the Cochrane RoB 2 tool. Green, yellow, and red indicate low risk, some concerns, and high risk of bias, respectively.

**Figures S3-S18. Leave-one-out sensitivity analyses.**

| Study | SMD | 95%-CI | P-value | Tau2 | Tau | I2 |
|---|---|---|---|---|---|---|
| Omitting Boer et al.2014 | 1.24 | [0.97; 1.50] | < 0.0001 | 1.5856 | 1.2592 | 84.0% |
| Omitting Chuensiri et al.2018 | 1.10 | [0.85; 1.36] | < 0.0001 | 0.7547 | 0.8688 | 77.1% |
| Omitting Lazzer et al.2017 | 1.31 | [1.05; 1.57] | < 0.0001 | 1.2923 | 1.1368 | 81.4% |
| Omitting Martin et al.2015 | 1.21 | [0.94; 1.48] | < 0.0001 | 1.6101 | 1.2689 | 84.2% |
| Omitting Martin-Smith et al.2019 | 1.11 | [0.84; 1.39] | < 0.0001 | 1.6178 | 1.2719 | 83.7% |
| Omitting Racil et al.2013 | 1.14 | [0.89; 1.40] | < 0.0001 | 1.5657 | 1.2513 | 83.6% |
| Omitting Rosenkranz et al.2012 | 1.18 | [0.92; 1.44] | < 0.0001 | 1.6010 | 1.2653 | 84.2% |
| Omitting Salus et al.2022(a) | 1.10 | [0.84; 1.36] | < 0.0001 | 1.3592 | 1.1658 | 81.7% |
| Omitting Salus et al.2022(b) | 1.10 | [0.84; 1.36] | < 0.0001 | 1.3592 | 1.1658 | 81.7% |
| Omitting Su et al.2025 | 1.33 | [1.06; 1.59] | < 0.0001 | 1.3360 | 1.1558 | 81.3% |
| Omitting Taylor et al.2019 | 1.25 | [0.98; 1.51] | < 0.0001 | 1.5710 | 1.2534 | 83.9% |
| Omitting Williams et al.2022 | 1.26 | [1.00; 1.52] | < 0.0001 | 1.4544 | 1.2060 | 83.3% |
| **Common effect model** | **1.19** | **[0.94; 1.45]** | **< 0.0001** | **1.4129** | **1.1886** | **82.6%** |

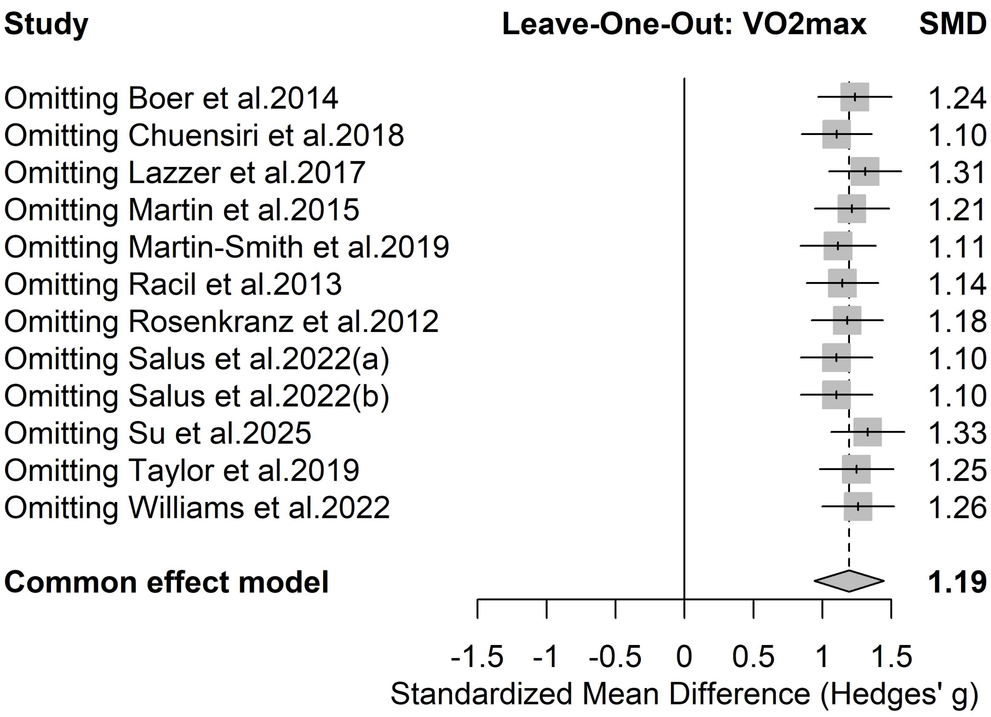

| Study | SMD | 95%-CI | P-value | Tau2 | Tau | I2 |
|---|---|---|---|---|---|---|
| Omitting Boer et al.2014 | -0.41 | [-0.73; -0.08] | 0.0135 | 3.5067 | 1.8726 | 92.2% |
| Omitting Chuensiri et al.2018 | -0.20 | [-0.50; 0.11] | 0.2024 | 1.6221 | 1.2736 | 88.3% |
| Omitting Martin-Smith et al.2019 | -0.09 | [-0.41; 0.24] | 0.6037 | 3.2672 | 1.8075 | 90.2% |
| Omitting Racil et al.2013 | -0.39 | [-0.71; -0.08] | 0.0146 | 3.5029 | 1.8716 | 92.2% |
| Omitting Rosenkranz et al.2012 | -0.28 | [-0.58; 0.03] | 0.0747 | 3.2528 | 1.8036 | 91.7% |
| Omitting Salus et al.2022(a) | -0.63 | [-0.94; -0.32] | < 0.0001 | 1.7256 | 1.3136 | 86.6% |
| Omitting Su et al.2025 | -0.43 | [-0.75; -0.11] | 0.0083 | 3.4633 | 1.8610 | 92.1% |
| Omitting Tadiotto et al.2023 | -0.51 | [-0.84; -0.18] | 0.0024 | 3.4065 | 1.8457 | 91.8% |
| **Common effect model** | **-0.37** | **[-0.66; -0.07]** | **0.0154** | **2.9507** | **1.7178** | **91.0%** |

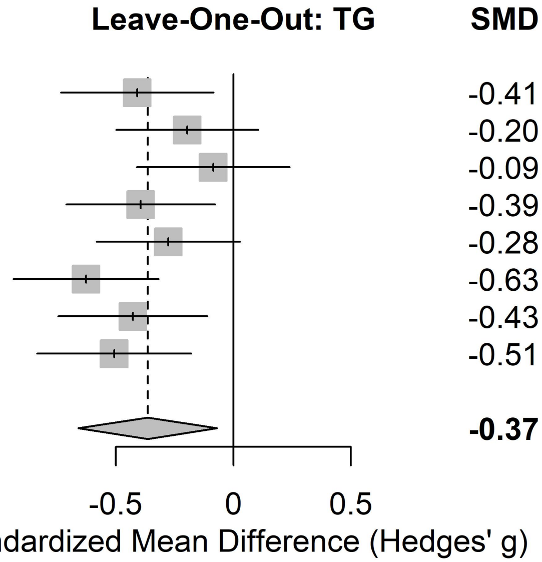

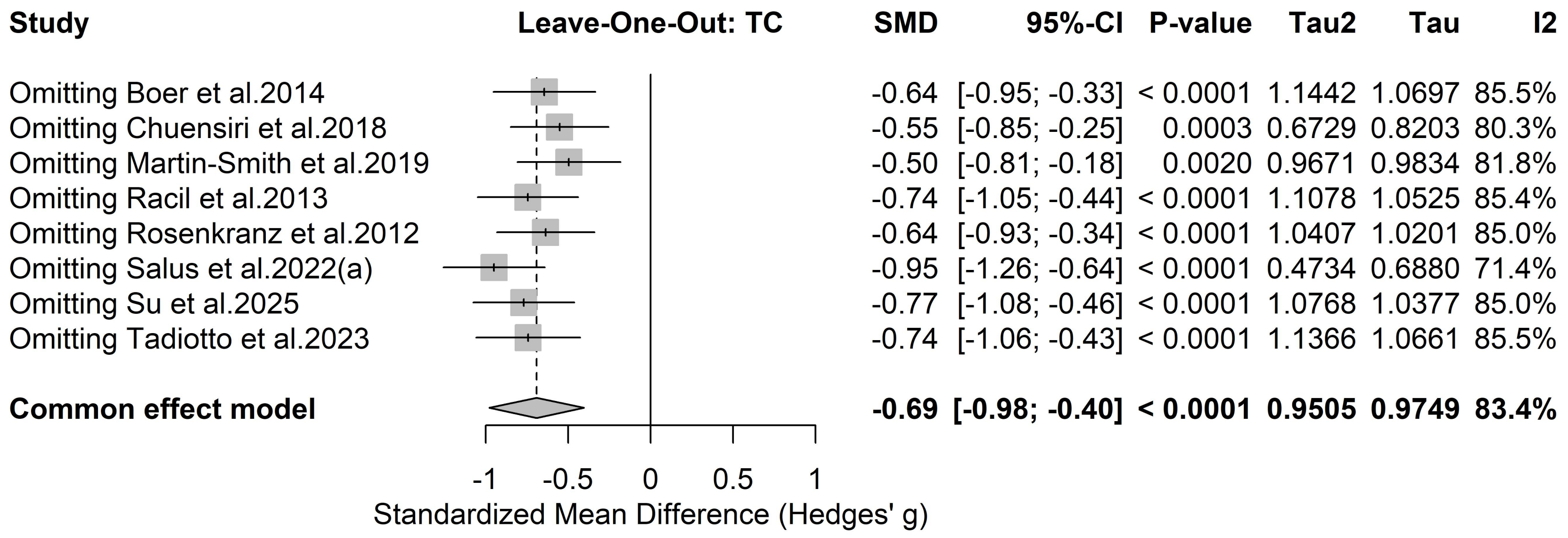
Study
Leave-One-Out: TC
SMD 95%-CI P-value Tau2 Tau I2
Omitting Boer et al.2014 -0.64 [-0.95; -0.33] < 0.0001 1.1442 1.0697 85.5%
Omitting Chuensiri et al.2018 -0.55 [-0.85; -0.25] 0.0003 0.6729 0.8203 80.3%
Omitting Martin-Smith et al.2019 -0.50 [-0.81; -0.18] 0.0020 0.9671 0.9834 81.8%
Omitting Racil et al.2013 -0.74 [-1.05; -0.44] < 0.0001 1.1078 1.0525 85.4%
Omitting Rosenkranz et al.2012 -0.64 [-0.93; -0.34] < 0.0001 1.0407 1.0201 85.0%
Omitting Salus et al.2022(a) -0.95 [-1.26; -0.64] < 0.0001 0.4734 0.6880 71.4%
Omitting Su et al.2025 -0.77 [-1.08; -0.46] < 0.0001 1.0768 1.0377 85.0%
Omitting Tadiotto et al.2023 -0.74 [-1.06; -0.43] < 0.0001 1.1366 1.0661 85.5%
Common effect model -0.69 [-0.98; -0.40] < 0.0001 0.9505 0.9749 83.4%
-1 -0.5 0 0.5 1
Standardized Mean Difference (Hedges' g)

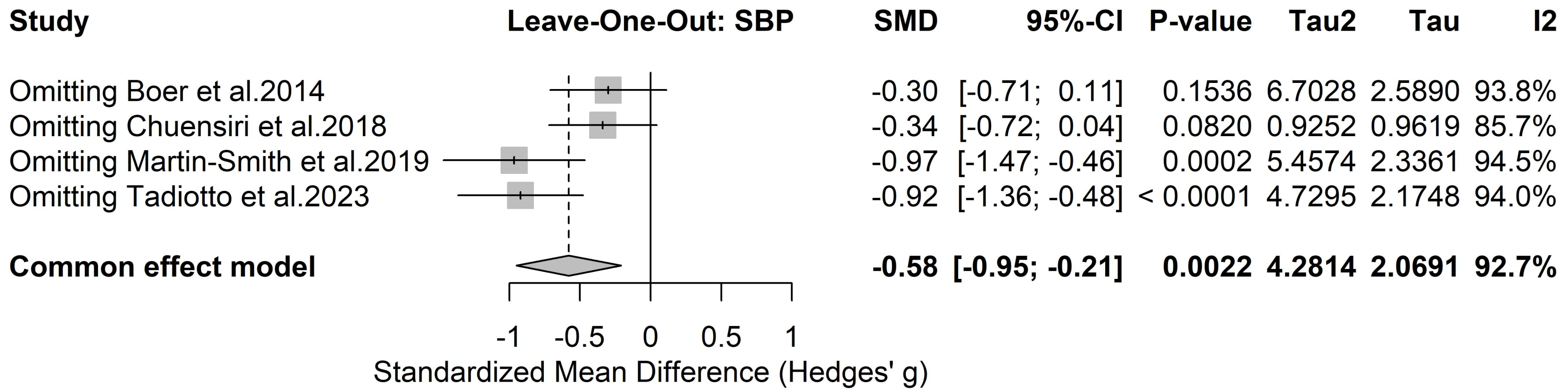
Study
Leave-One-Out: SBP
SMD 95%-CI P-value Tau2 Tau I2
Omitting Boer et al.2014 -0.30 [-0.71; 0.11] 0.1536 6.7028 2.5890 93.8%
Omitting Chuensiri et al.2018 -0.34 [-0.72; 0.04] 0.0820 0.9252 0.9619 85.7%
Omitting Martin-Smith et al.2019 -0.97 [-1.47; -0.46] 0.0002 5.4574 2.3361 94.5%
Omitting Tadiotto et al.2023 -0.92 [-1.36; -0.48] < 0.0001 4.7295 2.1748 94.0%
Common effect model -0.58 [-0.95; -0.21] 0.0022 4.2814 2.0691 92.7%
-1 -0.5 0 0.5 1
Standardized Mean Difference (Hedges' g)

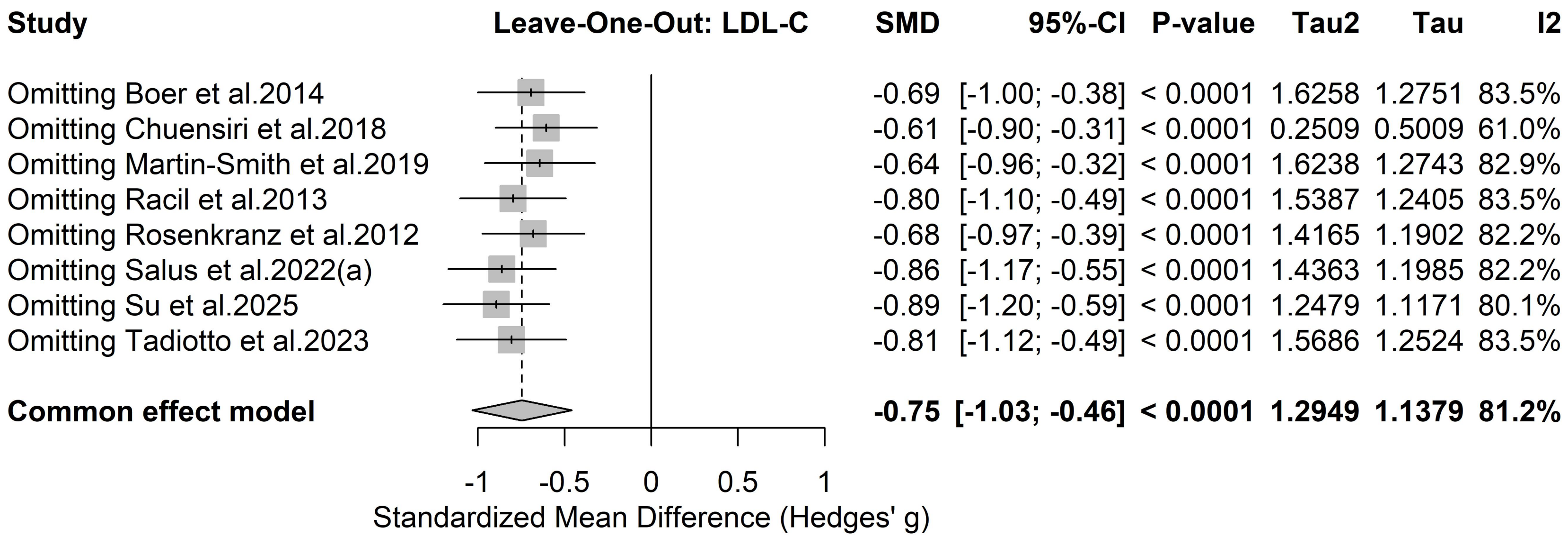
Study
Leave-One-Out: LDL-C
SMD
95%-CI
P-value
Tau2
Tau
I2
Omitting Boer et al.2014 -0.69 [-1.00; -0.38] < 0.0001 1.6258 1.2751 83.5%
Omitting Chuensiri et al.2018 -0.61 [-0.90; -0.31] < 0.0001 0.2509 0.5009 61.0%
Omitting Martin-Smith et al.2019 -0.64 [-0.96; -0.32] < 0.0001 1.6238 1.2743 82.9%
Omitting Racil et al.2013 -0.80 [-1.10; -0.49] < 0.0001 1.5387 1.2405 83.5%
Omitting Rosenkranz et al.2012 -0.68 [-0.97; -0.39] < 0.0001 1.4165 1.1902 82.2%
Omitting Salus et al.2022(a) -0.86 [-1.17; -0.55] < 0.0001 1.4363 1.1985 82.2%
Omitting Su et al.2025 -0.89 [-1.20; -0.59] < 0.0001 1.2479 1.1171 80.1%
Omitting Tadiotto et al.2023 -0.81 [-1.12; -0.49] < 0.0001 1.5686 1.2524 83.5%
Common effect model -0.75 [-1.03; -0.46] < 0.0001 1.2949 1.1379 81.2%
-1
-0.5
0
0.5
1
Standardized Mean Difference (Hedges' g)

| Study | Leave-One-Out: Insulin | SMD | 95%-CI | P-value | Tau2 | Tau | I2 |
|---|---|---|---|---|---|---|---|
| Omitting Boer et al.2014 | | -0.34 | [-0.66; -0.03] | 0.0342 | 1.8460 | 1.3587 | 88.9% |
| Omitting Martin et al.2015 | | -0.24 | [-0.55; 0.08] | 0.1439 | 1.4760 | 1.2149 | 85.3% |
| Omitting Martin-Smith et al.2019 | | -0.68 | [-1.01; -0.34] | < 0.0001 | 1.7404 | 1.3192 | 88.6% |
| Omitting Racil et al.2013 | | -0.32 | [-0.63; -0.02] | 0.0365 | 0.8810 | 0.9386 | 84.7% |
| Omitting Salus et al.2022(a) | | -0.67 | [-0.99; -0.35] | < 0.0001 | 1.4587 | 1.2077 | 87.4% |
| Omitting Tadiotto et al.2023 | | -0.54 | [-0.86; -0.21] | 0.0013 | 1.8733 | 1.3687 | 89.7% |
| Omitting Williams et al.2022 | | -0.55 | [-0.86; -0.25] | 0.0004 | 1.6549 | 1.2864 | 89.2% |
| **Common effect model** | | **-0.47** | **[-0.77; -0.18]** | **0.0017** | **1.5455** | **1.2432** | **87.9%** |

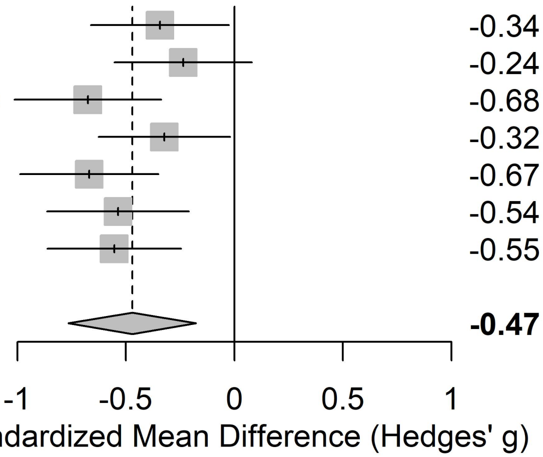

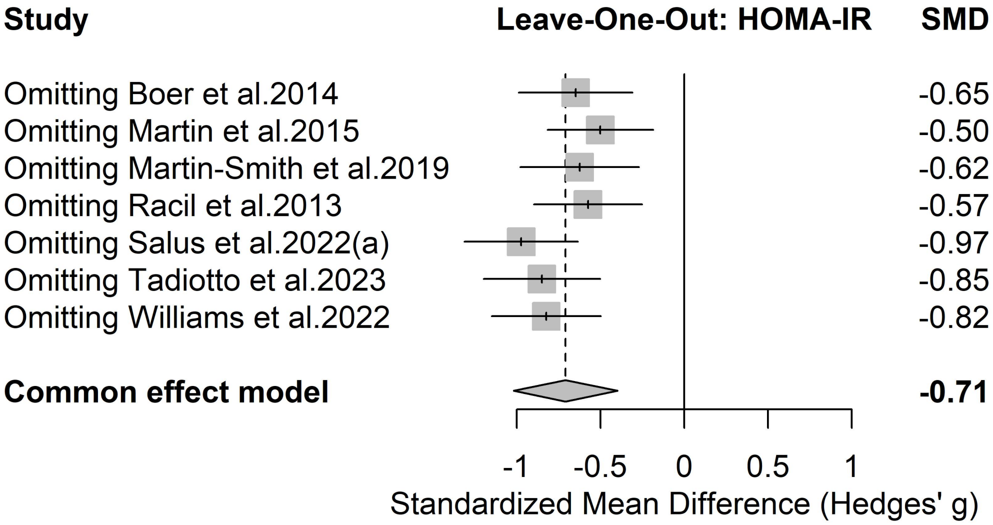


| Study | SMD | 95%-CI | P-value | Tau2 | Tau | I2 |
|---|---|---|---|---|---|---|
| Omitting Boer et al.2014 | -0.65 | [-0.99; -0.31] | 0.0002 | 5.6716 | 2.3815 | 92.9% |
| Omitting Martin et al.2015 | -0.50 | [-0.82; -0.19] | 0.0019 | 0.7786 | 0.8824 | 80.3% |
| Omitting Martin-Smith et al.2019 | -0.62 | [-0.98; -0.27] | 0.0006 | 5.6756 | 2.3824 | 92.9% |
| Omitting Racil et al.2013 | -0.57 | [-0.90; -0.25] | 0.0005 | 5.4505 | 2.3346 | 92.1% |
| Omitting Salus et al.2022(a) | -0.97 | [-1.31; -0.63] | < 0.0001 | 4.7458 | 2.1785 | 91.2% |
| Omitting Tadiotto et al.2023 | -0.85 | [-1.20; -0.50] | < 0.0001 | 5.3826 | 2.3200 | 92.6% |
| Omitting Williams et al.2022 | -0.82 | [-1.15; -0.50] | < 0.0001 | 5.0122 | 2.2388 | 92.4% |
| **Common effect model** | **-0.71** | **[-1.02; -0.40]** | **< 0.0001** | **4.5804** | **2.1402** | **91.5%** |

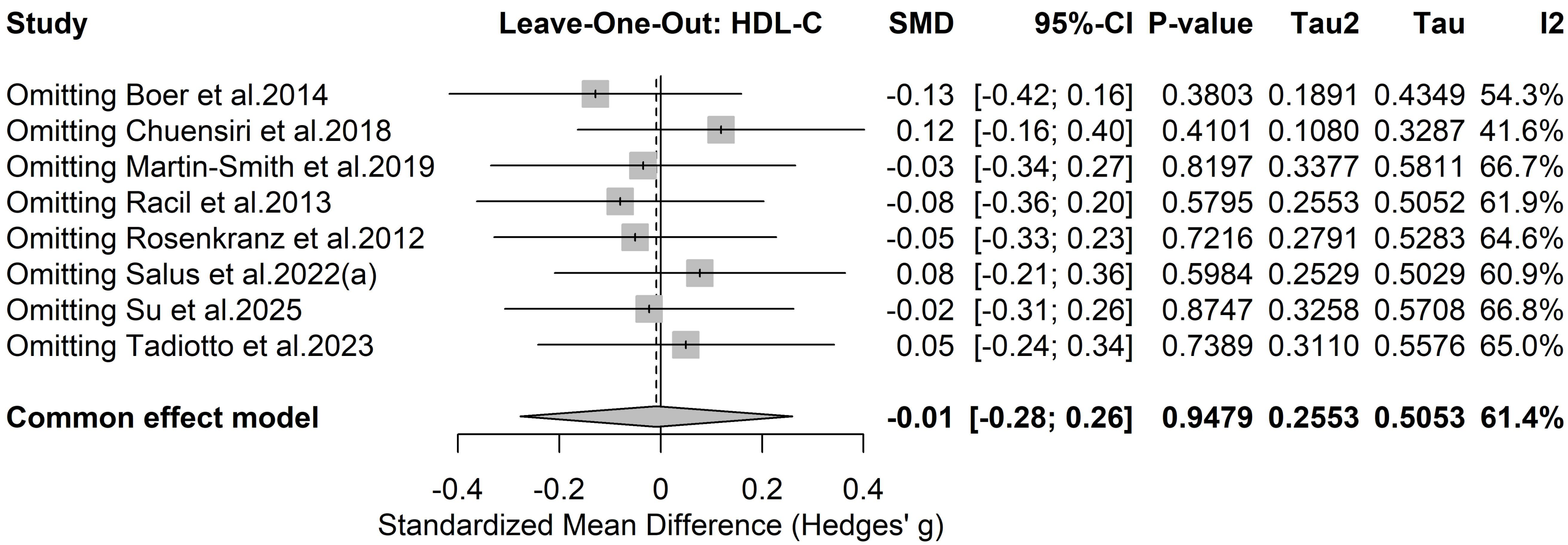
Study
Leave-One-Out: HDL-C
SMD
95%-CI
P-value
Tau2
Tau
I2
Omitting Boer et al.2014 -0.13 [-0.42; 0.16] 0.3803 0.1891 0.4349 54.3%
Omitting Chuensiri et al.2018 0.12 [-0.16; 0.40] 0.4101 0.1080 0.3287 41.6%
Omitting Martin-Smith et al.2019 -0.03 [-0.34; 0.27] 0.8197 0.3377 0.5811 66.7%
Omitting Racil et al.2013 -0.08 [-0.36; 0.20] 0.5795 0.2553 0.5052 61.9%
Omitting Rosenkranz et al.2012 -0.05 [-0.33; 0.23] 0.7216 0.2791 0.5283 64.6%
Omitting Salus et al.2022(a) 0.08 [-0.21; 0.36] 0.5984 0.2529 0.5029 60.9%
Omitting Su et al.2025 -0.02 [-0.31; 0.26] 0.8747 0.3258 0.5708 66.8%
Omitting Tadiotto et al.2023 0.05 [-0.24; 0.34] 0.7389 0.3110 0.5576 65.0%
Common effect model -0.01 [-0.28; 0.26] 0.9479 0.2553 0.5053 61.4%
-0.4
-0.2
0
0.2
0.4
Standardized Mean Difference (Hedges' g)

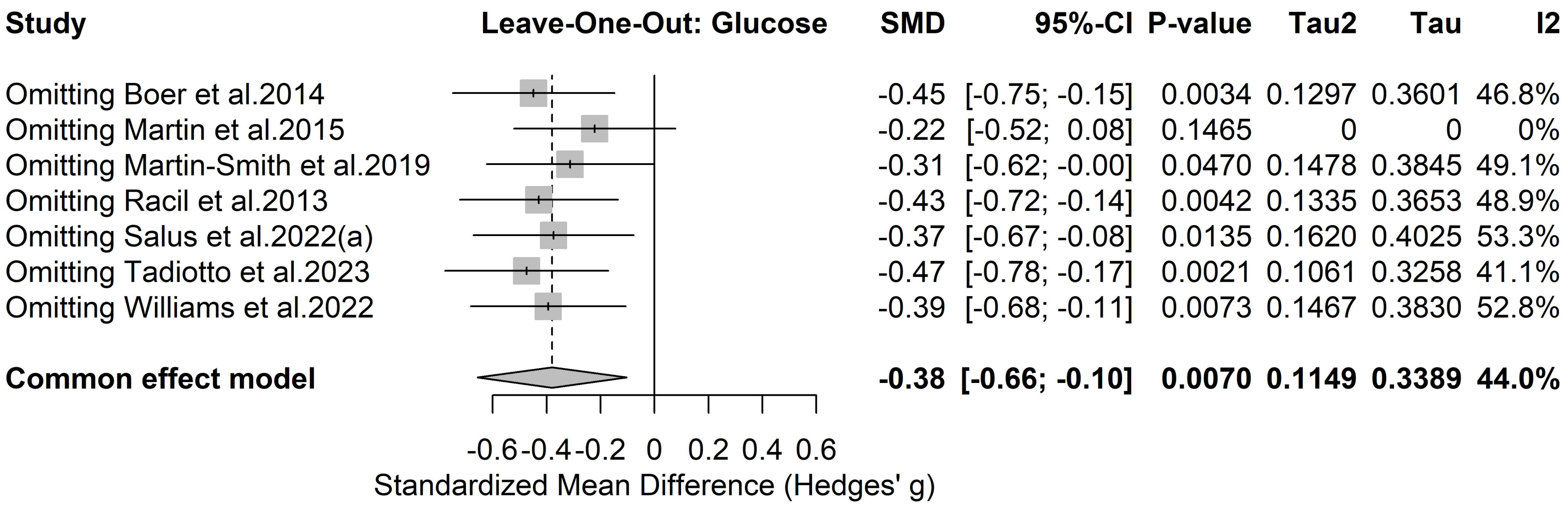
Study
Leave-One-Out: Glucose
SMD
95%-CI
P-value
Tau2
Tau
I2
Omitting Boer et al.2014 -0.45 [-0.75; -0.15] 0.0034 0.1297 0.3601 46.8%
Omitting Martin et al.2015 -0.22 [-0.52; 0.08] 0.1465 0 0 0%
Omitting Martin-Smith et al.2019 -0.31 [-0.62; -0.00] 0.0470 0.1478 0.3845 49.1%
Omitting Racil et al.2013 -0.43 [-0.72; -0.14] 0.0042 0.1335 0.3653 48.9%
Omitting Salus et al.2022(a) -0.37 [-0.67; -0.08] 0.0135 0.1620 0.4025 53.3%
Omitting Tadiotto et al.2023 -0.47 [-0.78; -0.17] 0.0021 0.1061 0.3258 41.1%
Omitting Williams et al.2022 -0.39 [-0.68; -0.11] 0.0073 0.1467 0.3830 52.8%
Common effect model -0.38 [-0.66; -0.10] 0.0070 0.1149 0.3389 44.0%
-0.6 -0.4 -0.2 0 0.2 0.4 0.6
Standardized Mean Difference (Hedges' g)

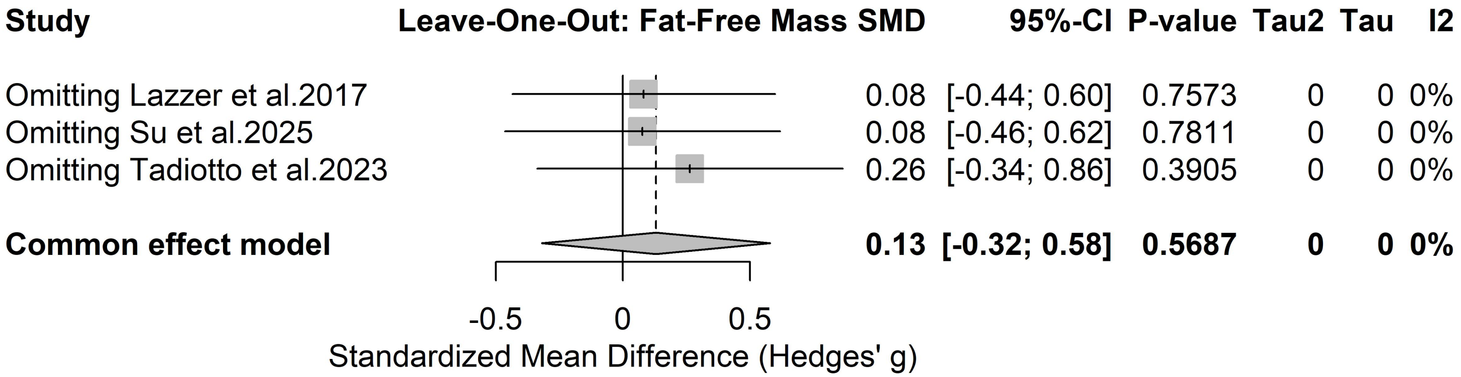
Study
Leave-One-Out: Fat-Free Mass
SMD
95%-CI
P-value
Tau2
Tau
I2
Omitting Lazzer et al.2017 0.08 [-0.44; 0.60] 0.7573 0 0 0%
Omitting Su et al.2025 0.08 [-0.46; 0.62] 0.7811 0 0 0%
Omitting Tadiotto et al.2023 0.26 [-0.34; 0.86] 0.3905 0 0 0%
Common effect model 0.13 [-0.32; 0.58] 0.5687 0 0 0%
-0.5 0 0.5
Standardized Mean Difference (Hedges' g)

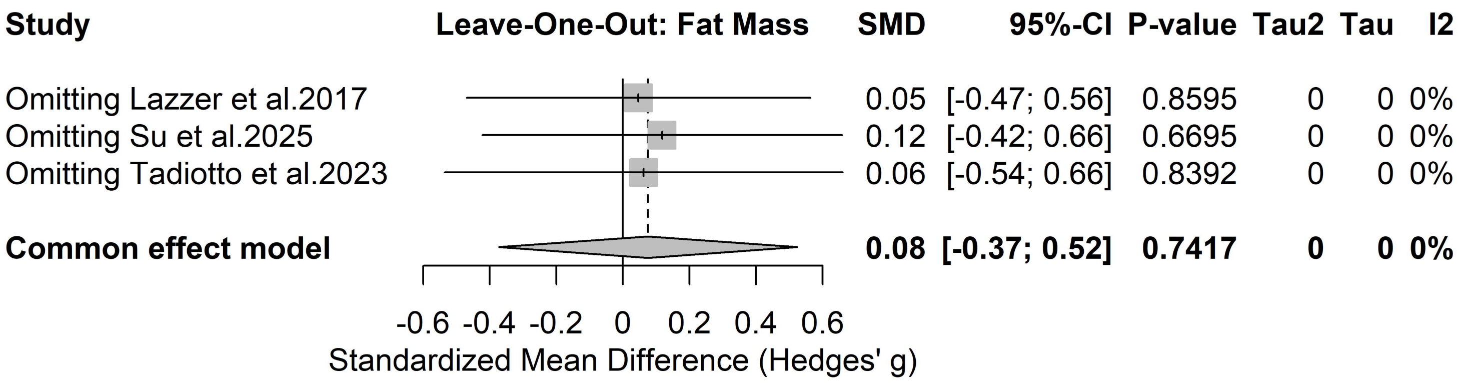
Study
Leave-One-Out: Fat Mass
SMD
95%-CI
P-value
Tau2
Tau
I2
Omitting Lazzer et al.2017 0.05 [-0.47; 0.56] 0.8595 0 0 0%
Omitting Su et al.2025 0.12 [-0.42; 0.66] 0.6695 0 0 0%
Omitting Tadiotto et al.2023 0.06 [-0.54; 0.66] 0.8392 0 0 0%
Common effect model 0.08 [-0.37; 0.52] 0.7417 0 0 0%
-0.6 -0.4 -0.2 0 0.2 0.4 0.6
Standardized Mean Difference (Hedges' g)

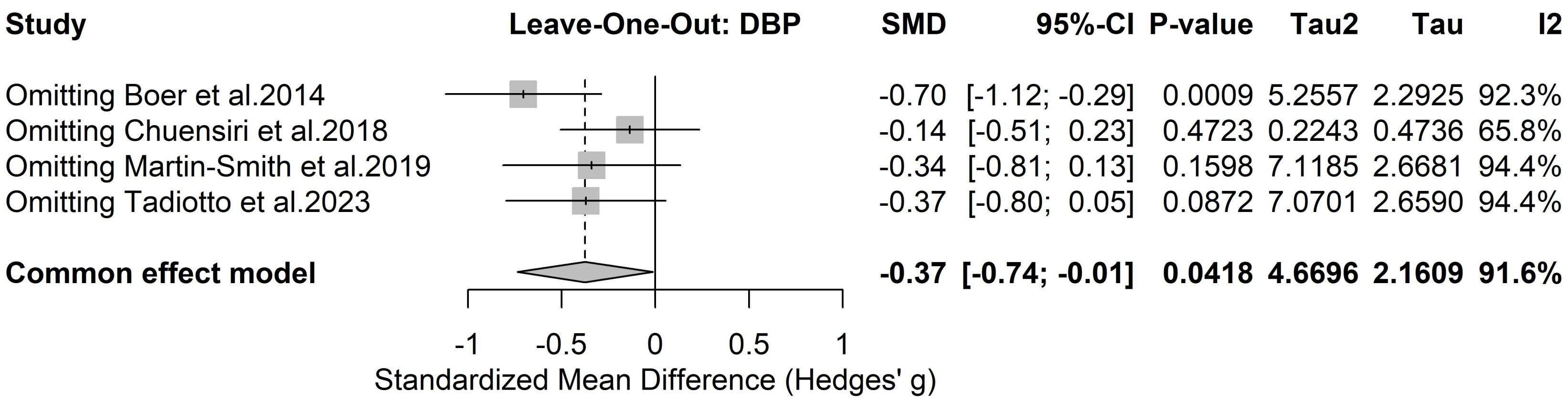
Study
Leave-One-Out: DBP
SMD
95%-CI
P-value
Tau2
Tau
I2
Omitting Boer et al.2014 -0.70 [-1.12; -0.29] 0.0009 5.2557 2.2925 92.3%
Omitting Chuensiri et al.2018 -0.14 [-0.51; 0.23] 0.4723 0.2243 0.4736 65.8%
Omitting Martin-Smith et al.2019 -0.34 [-0.81; 0.13] 0.1598 7.1185 2.6681 94.4%
Omitting Tadiotto et al.2023 -0.37 [-0.80; 0.05] 0.0872 7.0701 2.6590 94.4%
Common effect model -0.37 [-0.74; -0.01] 0.0418 4.6696 2.1609 91.6%
-1 -0.5 0 0.5 1
Standardized Mean Difference (Hedges' g)

| Study | SMD | 95%-CI | P-value | Tau2 | Tau | I2 |
|---|---|---|---|---|---|---|
| Omitting Boer et al.2014 | -0.16 | [-0.40; 0.08] | 0.1937 | 0 | 0 | 0% |
| Omitting Chuensiri et al.2018 | -0.16 | [-0.39; 0.08] | 0.1887 | 0 | 0 | 0% |
| Omitting Lau et al.2015 | -0.17 | [-0.40; 0.07] | 0.1644 | 0 | 0 | 0% |
| Omitting Lazzer et al.2017 | -0.18 | [-0.41; 0.06] | 0.1355 | 0 | 0 | 0% |
| Omitting Martin et al.2015 | -0.14 | [-0.38; 0.10] | 0.2531 | 0 | 0 | 0% |
| Omitting Racil et al.2013 | -0.12 | [-0.35; 0.12] | 0.3207 | 0 | 0 | 0% |
| Omitting Rosenkranz et al.2012 | -0.15 | [-0.38; 0.08] | 0.2133 | 0 | 0 | 0% |
| Omitting Salus et al.2022(a) | -0.16 | [-0.40; 0.08] | 0.1856 | 0 | 0 | 0% |
| Omitting Salus et al.2022(b) | -0.13 | [-0.37; 0.10] | 0.2732 | 0 | 0 | 0% |
| Omitting Su et al.2025 | -0.17 | [-0.40; 0.07] | 0.1586 | 0 | 0 | 0% |
| Omitting Taylor et al.2019 | -0.16 | [-0.40; 0.08] | 0.1876 | 0 | 0 | 0% |
| Omitting Williams et al.2022 | -0.16 | [-0.40; 0.07] | 0.1682 | 0 | 0 | 0% |
| **Common effect model** | **-0.15** | **[-0.38; 0.07]** | **0.1799** | **0** | **0** | **0%** |

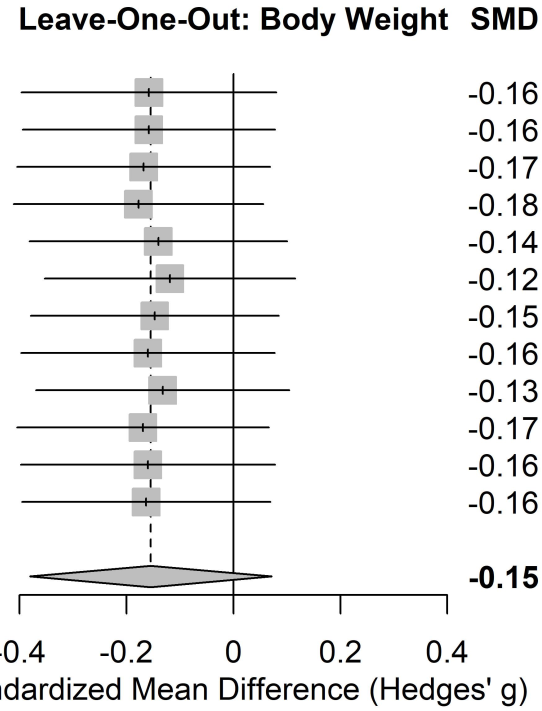

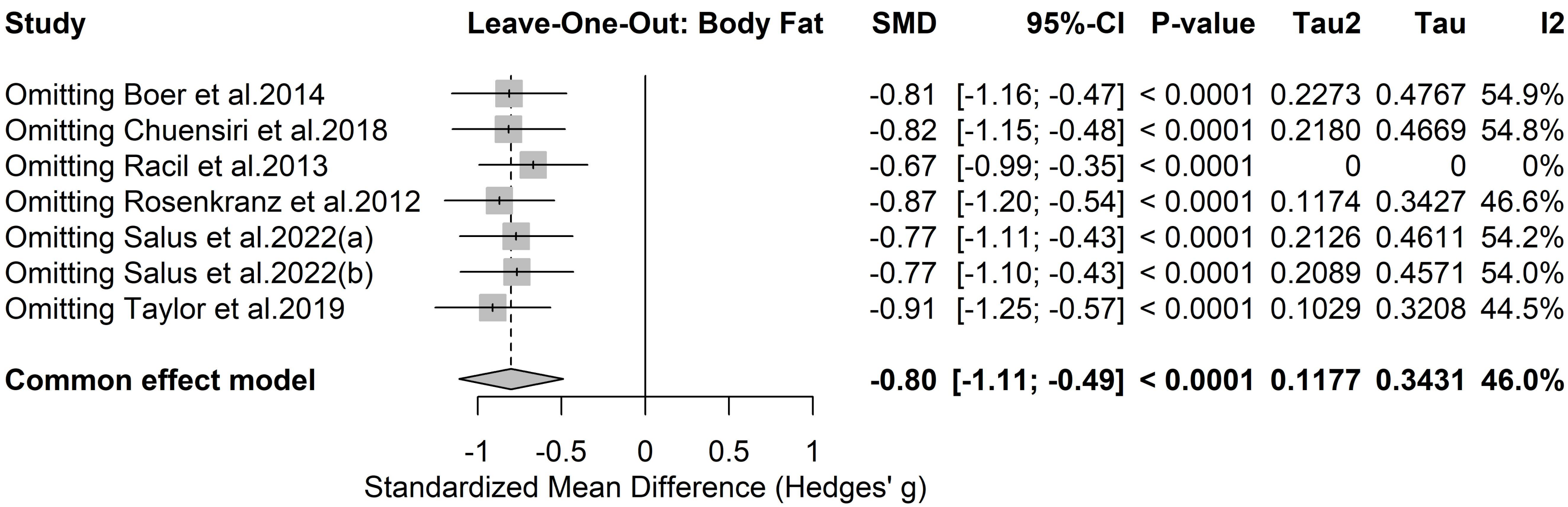
Study
Leave-One-Out: Body Fat
SMD
95%-CI
P-value
Tau2
Tau
I2
Omitting Boer et al.2014 -0.81 [-1.16; -0.47] < 0.0001 0.2273 0.4767 54.9%
Omitting Chuensiri et al.2018 -0.82 [-1.15; -0.48] < 0.0001 0.2180 0.4669 54.8%
Omitting Racil et al.2013 -0.67 [-0.99; -0.35] < 0.0001 0 0 0%
Omitting Rosenkranz et al.2012 -0.87 [-1.20; -0.54] < 0.0001 0.1174 0.3427 46.6%
Omitting Salus et al.2022(a) -0.77 [-1.11; -0.43] < 0.0001 0.2126 0.4611 54.2%
Omitting Salus et al.2022(b) -0.77 [-1.10; -0.43] < 0.0001 0.2089 0.4571 54.0%
Omitting Taylor et al.2019 -0.91 [-1.25; -0.57] < 0.0001 0.1029 0.3208 44.5%
Common effect model -0.80 [-1.11; -0.49] < 0.0001 0.1177 0.3431 46.0%
-1
-0.5
0
0.5
1
Standardized Mean Difference (Hedges' g)

frontiers

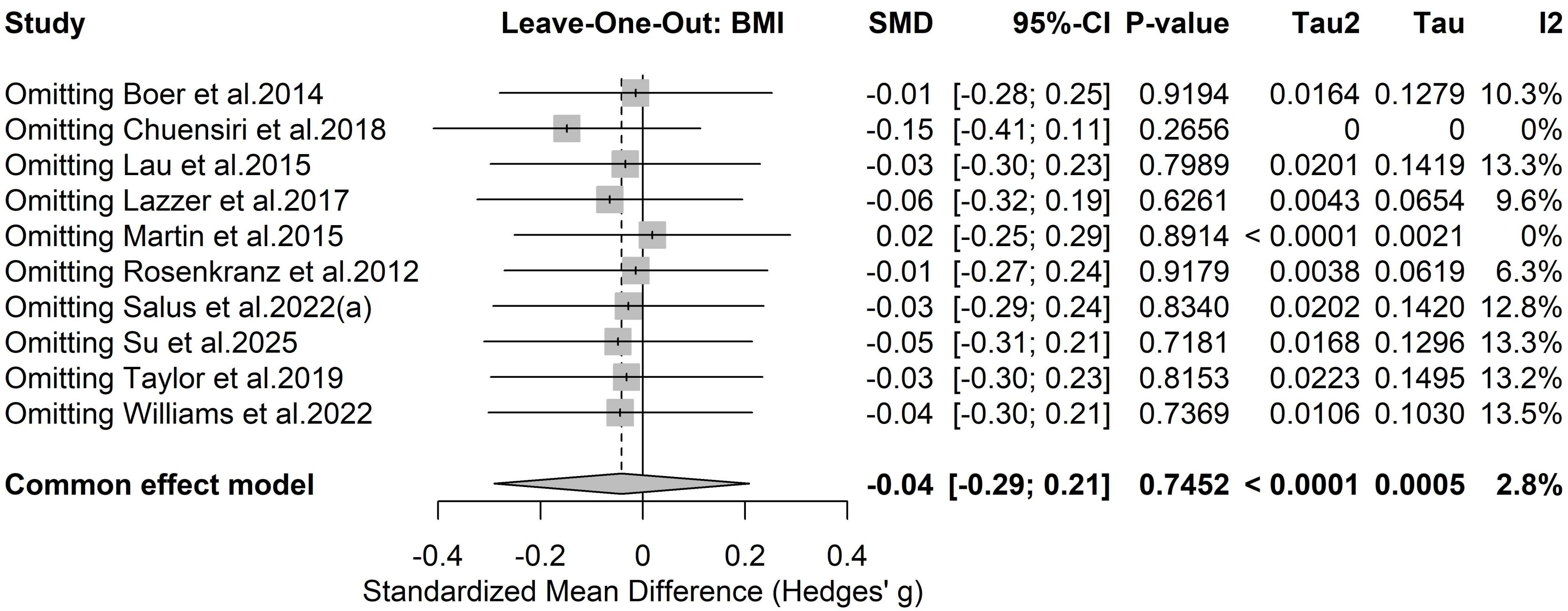
Study
Leave-One-Out: BMI
SMD
95%-CI
P-value
Tau2
Tau
I2
Omitting Boer et al.2014 -0.01 [-0.28; 0.25] 0.9194 0.0164 0.1279 10.3%
Omitting Chuensiri et al.2018 -0.15 [-0.41; 0.11] 0.2656 0 0 0%
Omitting Lau et al.2015 -0.03 [-0.30; 0.23] 0.7989 0.0201 0.1419 13.3%
Omitting Lazzer et al.2017 -0.06 [-0.32; 0.19] 0.6261 0.0043 0.0654 9.6%
Omitting Martin et al.2015 0.02 [-0.25; 0.29] 0.8914 < 0.0001 0.0021 0%
Omitting Rosenkranz et al.2012 -0.01 [-0.27; 0.24] 0.9179 0.0038 0.0619 6.3%
Omitting Salus et al.2022(a) -0.03 [-0.29; 0.24] 0.8340 0.0202 0.1420 12.8%
Omitting Su et al.2025 -0.05 [-0.31; 0.21] 0.7181 0.0168 0.1296 13.3%
Omitting Taylor et al.2019 -0.03 [-0.30; 0.23] 0.8153 0.0223 0.1495 13.2%
Omitting Williams et al.2022 -0.04 [-0.30; 0.21] 0.7369 0.0106 0.1030 13.5%
Common effect model -0.04 [-0.29; 0.21] 0.7452 < 0.0001 0.0005 2.8%
-0.4
-0.2
0
0.2
0.4
Standardized Mean Difference (Hedges' g)

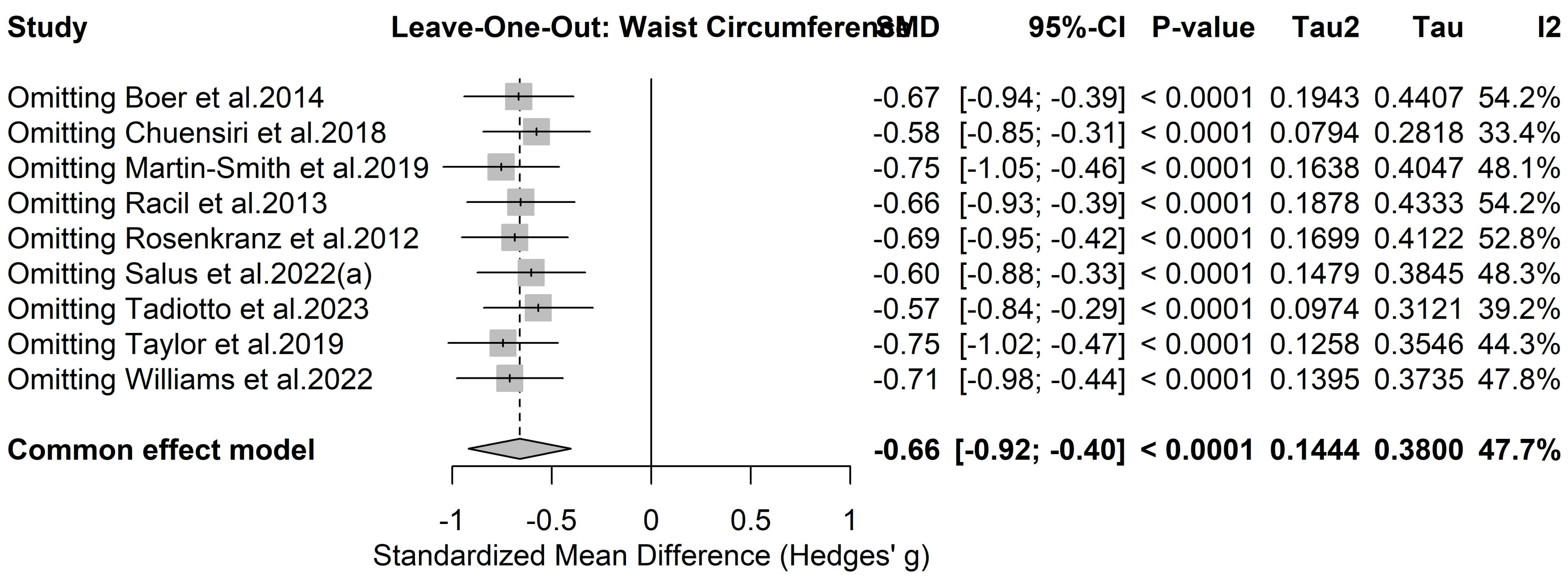


**Figures S3-S18. Leave-one-out sensitivity analyses.** Each plot corresponds to a specific outcome. Each point and horizontal line represents the pooled mean difference and 95% confidence interval after omitting the study listed on the y-axis. This analysis assesses the influence of individual studies on the overall pooled estimate.

**Figures S19-S21. Funnel plots for the assessment of publication bias.**

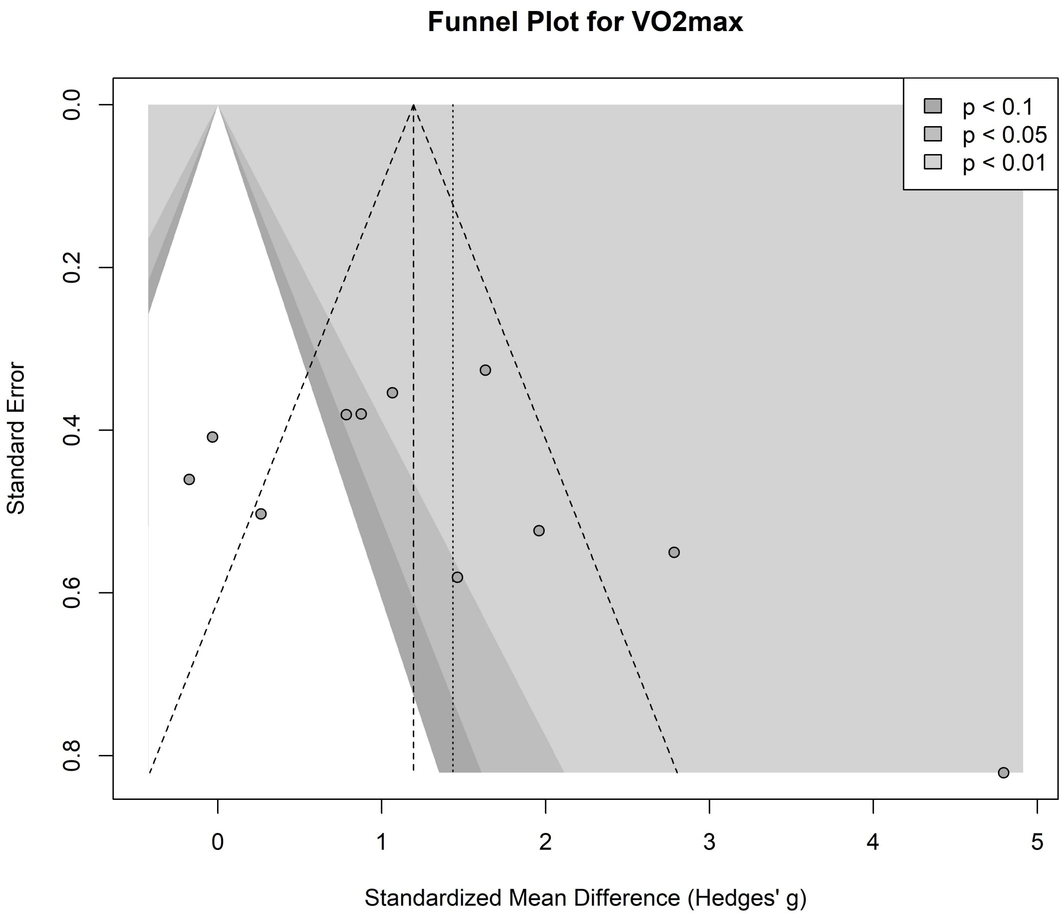

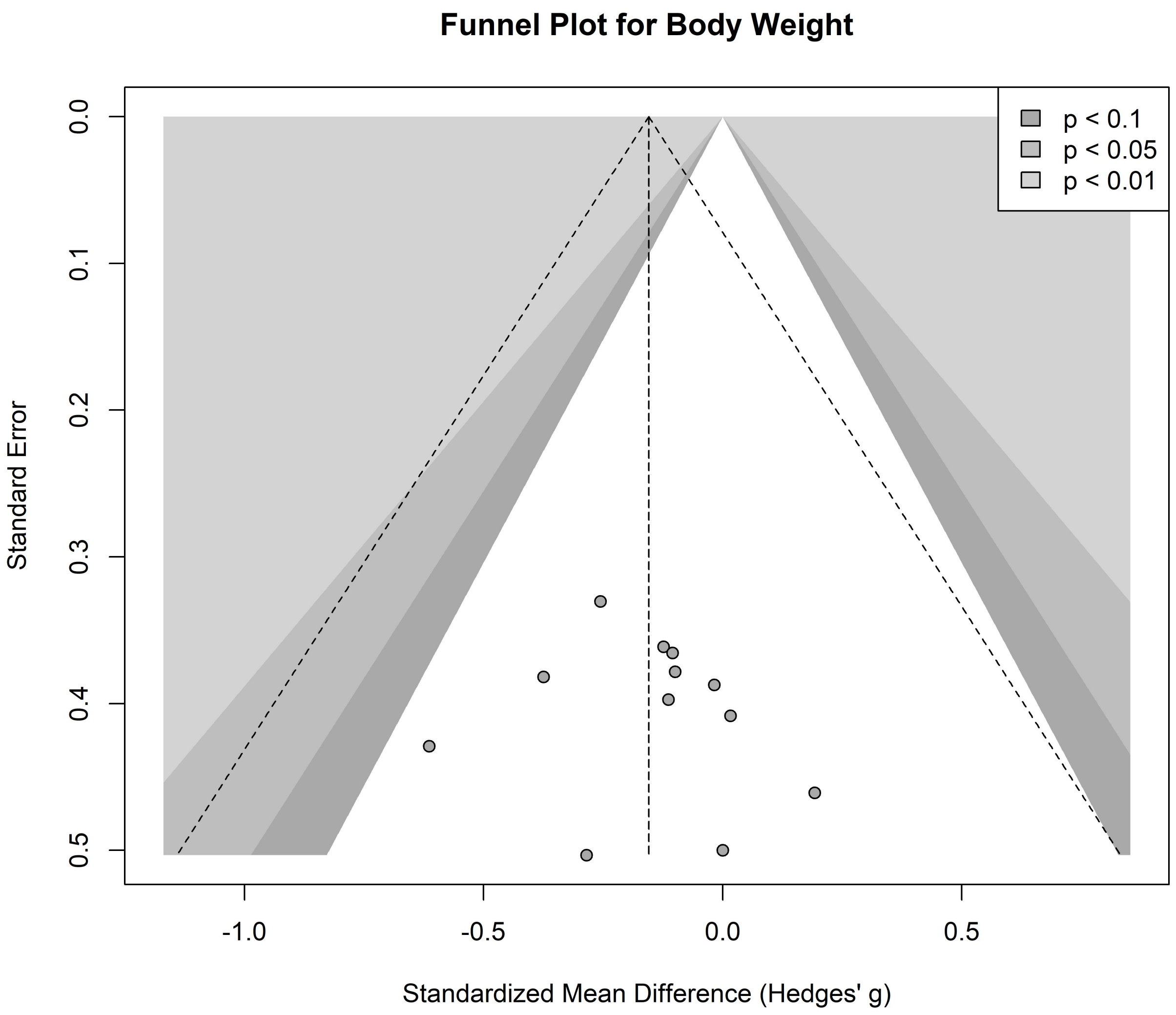
Funnel Plot for Body Weight
p < 0.1
p < 0.05
p < 0.01
Standard Error
0.0
0.1
0.2
0.3
0.4
0.5
-1.0
-0.5
0.0
0.5
Standardized Mean Difference (Hedges' g)

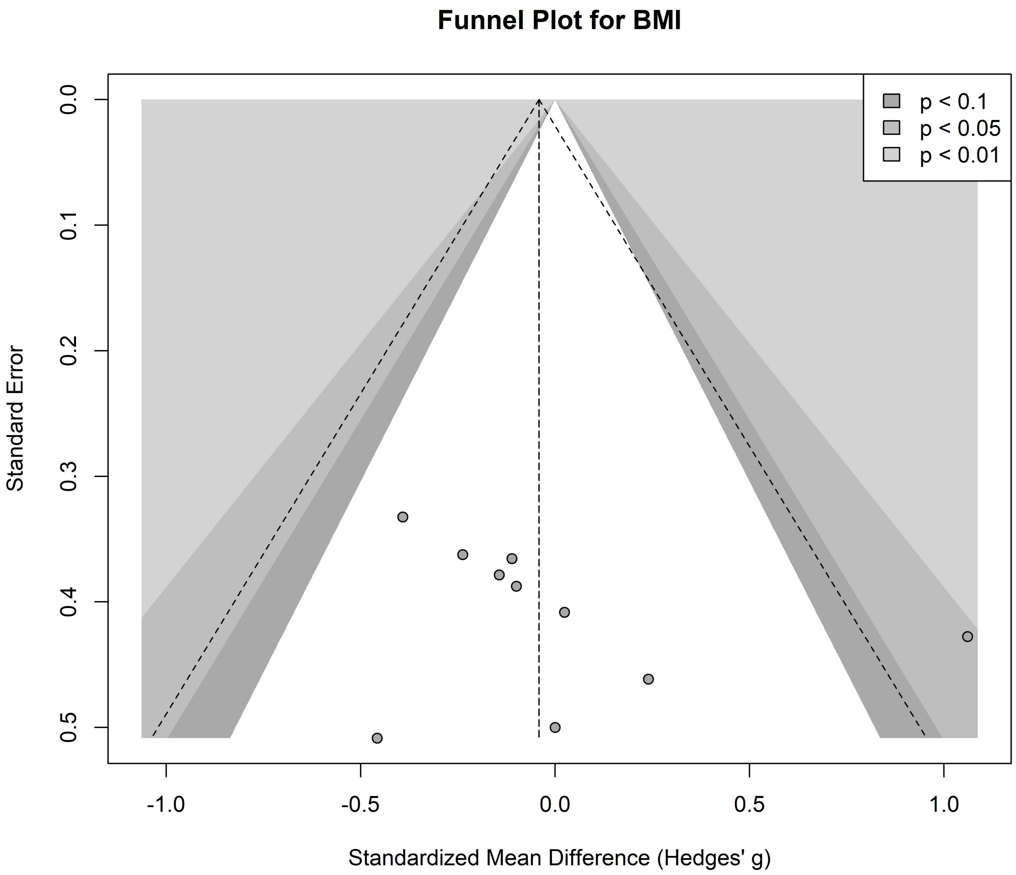


**Figures S19-S21. Funnel plots for the assessment of publication bias.** Each plot displays the effect estimate of individual studies (x-axis) against their standard error (y-axis). A symmetrical distribution of studies around the summary effect estimate (vertical line) suggests a low risk of publication bias.
